# Kaposi's Sarcoma Associated Herpesvirus Tegument Protein ORF75 Is Essential for Viral Lytic Replication and Plays a Critical Role in the Antagonization of ND10-Instituted Intrinsic Immunity

**DOI:** 10.1371/journal.ppat.1003863

**Published:** 2014-01-16

**Authors:** Florian Full, Doris Jungnickl, Nina Reuter, Elke Bogner, Kevin Brulois, Brigitte Scholz, Michael Stürzl, Jinjong Myoung, Jae U. Jung, Thomas Stamminger, Armin Ensser

**Affiliations:** 1 Institute for Clinical and Molecular Virology, Universitätsklinikum Erlangen, Friedrich-Alexander-Universität Erlangen-Nürnberg, Erlangen, Germany; 2 Institut für Medizinische Virologie, Charité-Universitätsmedizin Berlin, Berlin, Germany; 3 Department of Molecular Microbiology and Immunology, Keck School of Medicine, University of Southern California, Los Angeles, California, United States of America; 4 Division of Molecular and Experimental Surgery, Universitätsklinikum Erlangen, Friedrich-Alexander-Universität Erlangen-Nürnberg, Erlangen, Germany; 5 Novartis Institutes for Biomedical Research, Emeryville, California, United States of America; Florida State University, United States of America

## Abstract

Nuclear domain 10 (ND10) components are restriction factors that inhibit herpesviral replication. Effector proteins of different herpesviruses can antagonize this restriction by a variety of strategies, including degradation or relocalization of ND10 proteins. We investigated the interplay of Kaposi's Sarcoma-Associated Herpesvirus (KSHV) infection and cellular defense by nuclear domain 10 (ND10) components. Knock-down experiments in primary human cells show that KSHV-infection is restricted by the ND10 components PML and Sp100, but not by ATRX. After KSHV infection, ATRX is efficiently depleted and Daxx is dispersed from ND10, indicating that these two ND10 components can be antagonized by KSHV. We then identified the ORF75 tegument protein of KSHV as the viral factor that induces the disappearance of ATRX and relocalization of Daxx. ORF75 belongs to a viral protein family (viral FGARATs) that has homologous proteins in all gamma-herpesviruses. Isolated expression of ORF75 in primary cells induces a relocalization of PML and dispersal of Sp100, indicating that this viral effector protein is able to influence multiple ND10 components. Moreover, by constructing a KSHV mutant harboring a stop codon at the beginning of ORF75, we could demonstrate that ORF75 is absolutely essential for viral replication and the initiation of viral immediate-early gene expression. Using recombinant viruses either carrying Flag- or YFP-tagged variants of ORF75, we could further corroborate the role of ORF75 in the antagonization of ND10-mediated intrinsic immunity, and show that it is independent of the PML antagonist vIRF3. Members of the viral FGARAT family target different ND10 components, suggesting that the ND10 targets of viral FGARAT proteins have diversified during evolution. We assume that overcoming ND10 intrinsic defense constitutes a critical event in the replication of all herpesviruses; on the other hand, restriction of herpesviral replication by ND10 components may also promote latency as the default outcome of infection.

## Author Summary

Kaposi's Sarcoma-Associated Herpesvirus (KSHV) establishes a lifelong persistent infection in humans and is associated with tumors and lymphoproliferative disease, particularly upon immunosuppression. The virus has to overcome cellular intrinsic immunity in order to initiate viral protein expression and genome replication in primary infection. We demonstrated that KSHV is restricted by a cellular intrinsic immunity complex called nuclear domain 10 (ND10) and identified a critical role of the KSHV ORF75 protein, which is part of the viral particle, in this process. We found that ORF75 is essential for viral replication and that ORF75 leads to disappearance of the ND10 protein ATRX. Furthermore, it induces the relocalization of several other ND10 components. Noteworthy, all herpesviruses studied so far have evolved mechanisms for ND10 counteraction, indicating the importance of this step for herpesviral replication. The individual mechanisms, however, including the extent of ND10-antagonization, are of considerable variation between different herpesviruses. We speculate that, in contrast to efficient lytic replication of alphaherpesviruses, less effective ND10 counteraction may represent a doorway for gammaherpesviruses to latent infection.

## Introduction

Human Herpesvirus 8 (HHV8), or Kaposi's Sarcoma-Associated Herpesvirus (KSHV), belongs to the subfamily *Gammaherpesvirinae* and is grouped together with the closely related prototypic Saimiriine herpesvirus 2, or Herpesvirus saimiri (HVS), into the genus *Rhadinovirus*. KSHV was first discovered in 1994 from patients with Kaposi's Sarcoma (KS) lesions [1]. KSHV is the etiologic agent of three human diseases; a) KS, a skin tumor of endothelial origin, and two B cell malignancies, b) Primary Effusion Lymphoma and c) Multicentric Castleman's Disease. KS exists in several forms, classical KS, which is endemic in the Mediterranean and Middle East [2], African endemic KS, iatrogenic KS, which is associated with renal transplantation and AIDS-associated KS, which is among the leading causes of death in AIDS patients [3].

The promyelocytic leukemia protein (PML) is the main component of a subnuclear structure called nuclear domain 10 (ND10). ND10 components like PML, Sp100, Daxx and ATRX were identified as cellular restriction factors that are able to inhibit the replication of several DNA viruses, including herpesviruses. The restriction is assumed to be mediated by the recruitment of cellular proteins like heterochromatic protein 1 (HP1), DNA methyltransferases or histone deacetylases that induce a repressive chromatin status on the herpesviral genome [4]. This antiviral function of PML, however, is counteracted by most human herpesviruses through viral effector proteins using a variety of strategies, including degradation or relocalization of PML. So far, herpesviruses were mainly found to target the major ND10 component PML, as exemplified by the herpes simplex virus regulatory protein ICP0, which has been shown to induce the proteasomal degradation of PML either via acting as a SUMO-targeted ubiquitin ligase or via SUMO-independent ubiquitination [5]–[7]. Similarly, the IE1 protein of human cytomegalovirus targets PML via inducing a de-SUMOylation of this major ND10 component [8], [9]. Since SUMOylation of PML is essential for the integrity of ND10, this leads to a dispersal of the subnuclear structure. PML expression is induced by the interferon pathway [10], [11] and the protein is detected in high amounts in KSHV infected PEL cells [12]. KSHV vIRF3 (LANA2, K10.5) has been shown to colocalize with PML and its overexpression is associated with a redistribution of PML [13]. Another KSHV gene, ORF K8 encodes an early lytic protein, which shares homology to the EBV BZLF1 gene. K8 is a SUMO interaction motif (SIM) dependent, SUMO-2/3-specific SUMO E3 ligase [14] that colocalizes with PML [15]. K8 recruits p53 and probably sequesters it to PML bodies but has no function in dispersing PML bodies [12].

We recently demonstrated that infection of fibroblasts with HVS results in restriction of immediate-early gene expression, which can be alleviated by siRNA mediated knock down of PML [16]; PML is thus also a restriction factor for HVS infection. In contrast to all other Herpesviruses, HVS exclusively degrades the cellular ND10 component Sp100 whereas other factors like PML or Daxx remain intact. The ORF3 tegument protein of HVS was identified as being responsible for this effect. ORF3 induced the proteasomal degradation of Sp100, and a mutant HVS lacking the orf3-gene was no longer able to mediate Sp100 degradation [16].

The ORF3 of HVS shares homology with the cellular formylglycineamid-ribotideamidotransferase enzyme (FGARAT, EC 6.3.5.3). All gammaherpesviruses encode one to three FGARAT-homologous proteins in their genome. Interestingly, recent studies showed that ORF3-homologous proteins from murid gammaherpesvirus 68 (MHV-68) and from Epstein-Barr virus (EBV) modulate the ND10 proteins PML and ATRX, respectively, suggesting that the ND10 targets of the viral FGARAT protein family have diversified during evolution [17], [18]. The KSHV orf75 gene comprises one distinct member of the vFGARAT-family. The putative tegument protein ORF75 has been shown to be involved in NF-kB coactivation with KSHV K13/vFLIP [19].

This study addresses the role of ND10 components in the restriction of lytic replication of KSHV infection. We describe the essential protein ORF75 as a new viral effector, adding a new piece to the intricate puzzle of herpesvirus coevolution with cellular intrinsic immunity.

## Results

### KSHV infection is restricted by PML

**Figure 1:**
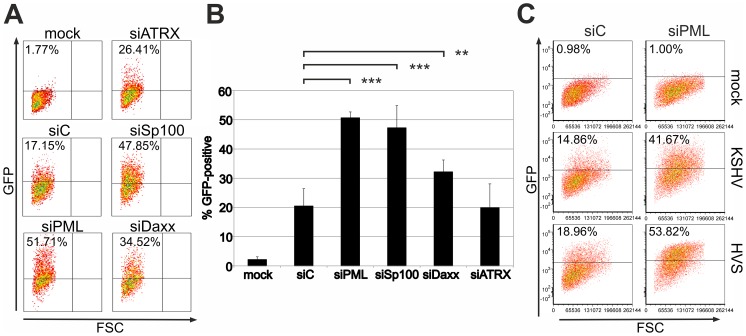
KSHV infection is restricted by PML and Sp100 but not ATRX. **A**: Equal numbers of HFF cells treated with siRNAs against ATRX (siATRX), PML (siPML), Sp100 (siSp100), Daxx (siDAXX) and control (siC), were infected with equal amounts of KSHV. GFP-expression was measured two days post infection by flow-cytometry. **B**: Mean value of at least 3 individual infection experiments according to A. Statistical analyses were conducted with GraphPad Prism 6 using the paired t test for siC and siSP100, siPML and siDAXX, respectively. **p<0.015; *** p<0.0001. **C**: Equal numbers of HFF cells treated with siRNAs against PML and control (siC) were infected with equal amounts of KSHV Bac16 wt and HVS Bac43 wt virus and the GFP-expression was measured two days post infection by flow-cytometry.

We recently demonstrated that PML acts as a restriction factor for infection with the gammaherpesvirus HVS [16]. To investigate whether ND10 components exert a similar effect on KSHV infection, we analyzed KSHV infection in primary human foreskin fibroblast (HFF) cells carrying an siRNA mediated knock-down of ND10 components. Using recombinant rKSHV.219 [20] we detected significantly more GFP-positive cells in the absence of PML (siPML) or Sp100 (siSp100) compared to control cells (siC) (Figure 1A and 1B). Moreover, we detected a moderate increase in GFP expression from the viral genome after knock-down of Daxx (siDaxx) compared to control cells. However, there was no enhancement of KSHV infection as indicated by GFP expression after depletion of ATRX (siATRX) (Figure 1A and 1B). KSHV-restriction was in a similar range as the restriction of HVS, suggesting that analogous mechanisms are active on these evolutionary closely related viruses (Figure 1C). Accordingly, ND10 components may compromise KSHV infection in primary human cells.

### KSHV infection leads to a disappearance of the ND10 component ATRX

**Figure 2:**
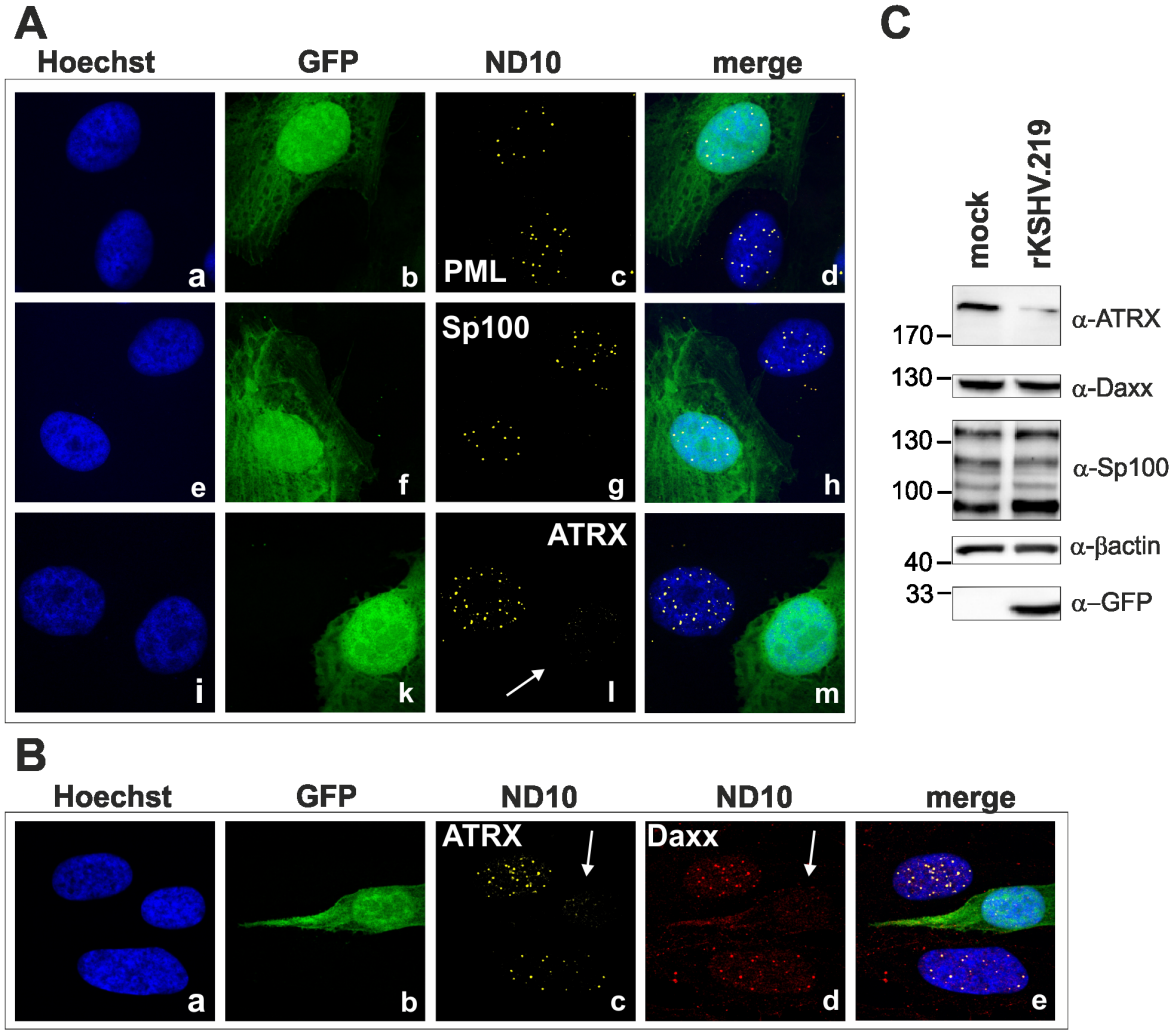
KSHV infection antagonizes the ND10 component ATRX. iSLK cells were seeded onto glass slides, infected with KSHV strain rKSHV.219 for 24**A**: single staining for PML (c), Sp100 (g) and ATRX (l) **B**: double staining for ATRX (c) and Daxx (d). Arrows indicate infected cells. **C**: iSLK cells were infected with KSHV strain rKSHV.219 with high MOI and harvested 20 h post infection. Whole cell lysates were prepared and expression of ND10 components analyzed by immunoblotting.

**Figure 3:**
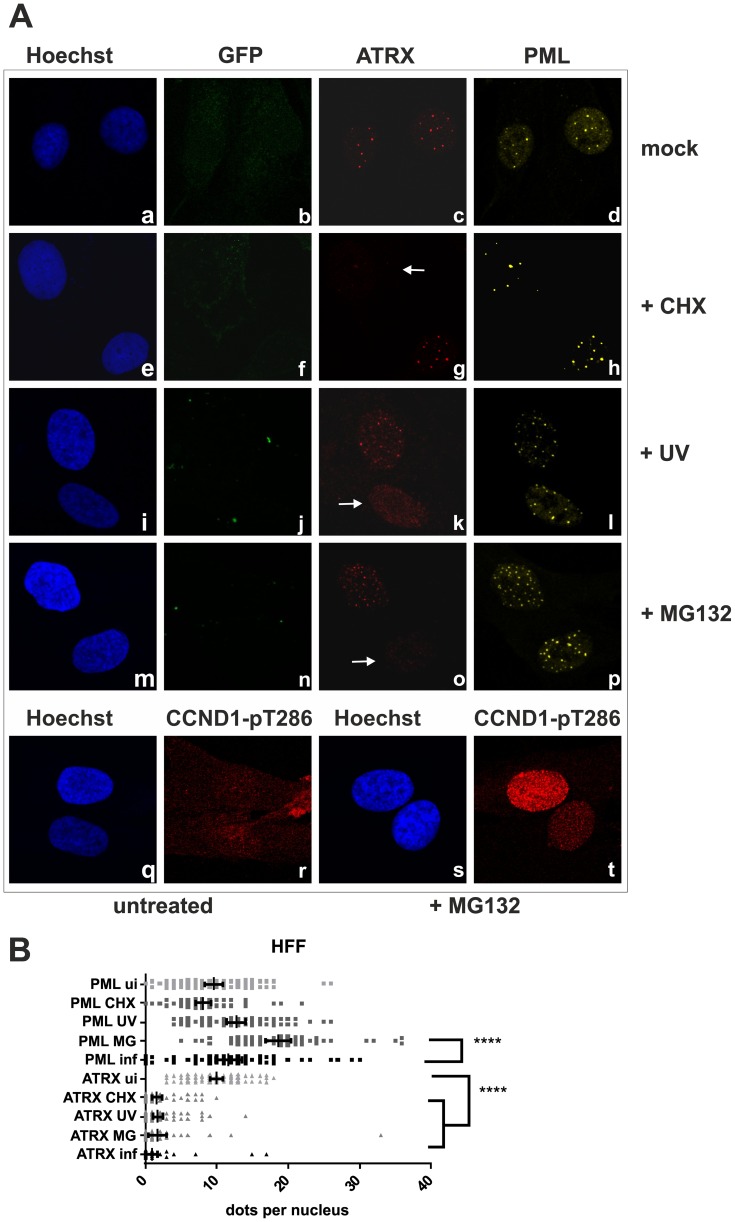
KSHV infection antagonizes the ND10 component ATRX independent of de novo protein synthesis. **A**: HFF cells were seeded onto glass slides, infected with KSHV strain rKSHV.219 or UV inactivated virus as indicated, along with exposure to the indicated chemicals o/n, and subjected to analysis of expression of different ND10 components ATRX and PML by immunofluorescence. Arrows indicate exemplary cells with relocalized ATRX. **B**: Quantitation of approx. 50 individual cells per each box by Fiji. Statistical analyses were conducted using the unpaired t test using GraphPad Prism 6 for Windows; **** P<0.0001.

We next wanted to elucidate the effect of KSHV *de novo* infection on major ND10 components. Therefore, epithelial iSLK cells, which promote lytic replication of KSHV [21], [22], were infected with rKSHV.219 and subjected to immunofluorescence analysis. In KSHV infected GFP positive iSLK cells, the dot like staining of ATRX characteristic for ND10 association was lost (Figure 2A). In addition, we observed a loss of dotted Daxx staining pattern in infected cells (Figure 2B). Using Western Blot experiments from iSLK cells infected with high multiplicity of infection (MOI), we could demonstrate that KSHV infection leads to a reduction of ATRX protein levels. However, this coincided only with a minimal decrease of Daxx levels in Western Blot experiments (Figure 2C). Both Western blot and immunofluorescence analysis revealed no effect of KSHV infection on the ND10 components PML and Sp100 in KSHV-infected cells, which were identified by GFP expression. PML and Sp100 were still detectable in numerous foci being characteristic for their assembly in ND10 structures (Figure 2). Thus, KSHV infection leads to the disappearance of ATRX and to the dispersion of Daxx from ND10; total Daxx protein in immunoblots is unchanged, however. In order to further corroborate that the observed effects are due to an incoming tegument protein, cells were infected in the presence of cycloheximide to inhibit de novo protein synthesis, or infected with UV-inactivated virus (Figure 3). ATRX also disappeared under both conditions, underlining that de novo protein synthesis is not necessary and the effect is caused by a viral tegument protein. In an additional set of experiments, treatment with the proteasome inhibitor MG-132 did also not reverse the effect of KSHV infection on ATRX, while the treatment stabilized the phosphorylated form of Cyclin D1 (CCND1-pT286) as expected (Figure 3A, lower panel).

### KSHV lytic induction leads to a dispersal of the ND10 components ATRX and Sp100

**Figure 4:**
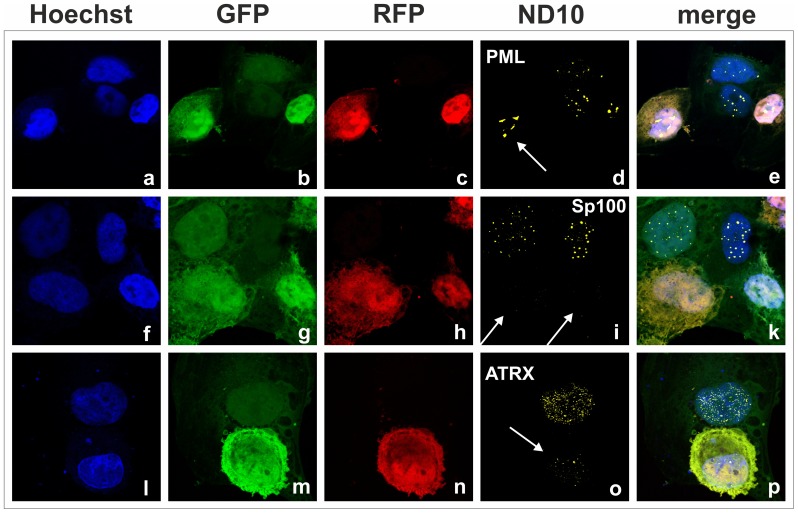
KSHV lytic replication leads to a loss of ATRX and Sp100 expression. iSLK.219 cells were seeded onto glass slides and lytic replication induced by adding sodium-butyrate and doxycyclin. Two days post induction, cells were harvested and subjected to analysis of expression of different ND10 components by immunofluorescence. Arrows indicate lytically replicating cells.

Next we wanted to know what happens after lytic induction of KSHV. Therefore we used epithelial iSLK.219 cells [21], [22], a cell line that harbors multiple copies of KSHV strain rKSHV.219 per cell. The KSHV strain rKSHV.219 expresses GFP under control of a constitutive EF1a promoter situated in the BACmid backbone, and red fluorescent protein (RFP) under control of the PAN promoter, which is switched on by the KSHV initiator of lytic viral replication RTA encoded by ORF50. Thus, infection per se results in GFP expression, and induction of early lytic gene expression can be easily monitored by RFP expression. Similar to our observation after *de novo* infection, KSHV lytic induction leads to a decrease of ATRX in cells expressing RFP (Figure 4, panel i to p); it is also able to overcome an apparent initial increase in ATRX expression after sodium-butyrate treatment of cells, which can be seen in cells with GFP-expression alone. Interestingly, we also observed the disappearance of Sp100 from ND10 in lytically infected cells Fig. 4, panels f to k). This indicates that a lytic gene product of KSHV may antagonize Sp100.

### KSHV orf75 induces relocalization and dispersion of multiple ND10 components

**Figure 5:**
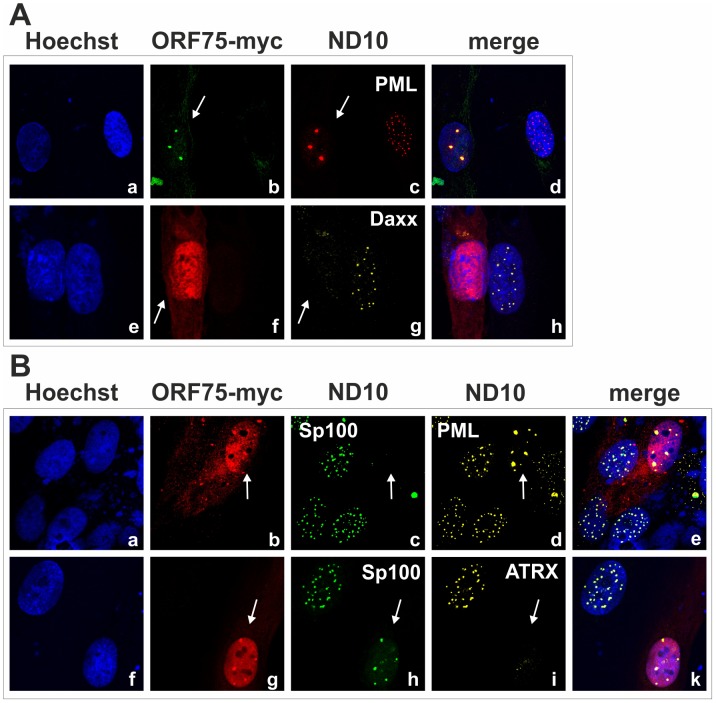
KSHV ORF75 is sufficient for antagonization of ATRX and Sp100. HFF cells were seeded onto glass slides and transfected with a vector encoding the full length ORF75 protein (pcDNA-orf75-myc). Two days post transfection, cells were harvested and subjected to analysis of expression of different ND10 components by immunofluorescence. **A**: staining for ORF75-myc and PML (b+c) and ORF75-myc and Daxx (f+g) **B**: triple staining for ORF75-myc and ND10 components using Ig-subtype specific secondary antibodies (Life Technologies). Arrows indicate transfected cells expressing ORF75.

**Figure 6:**
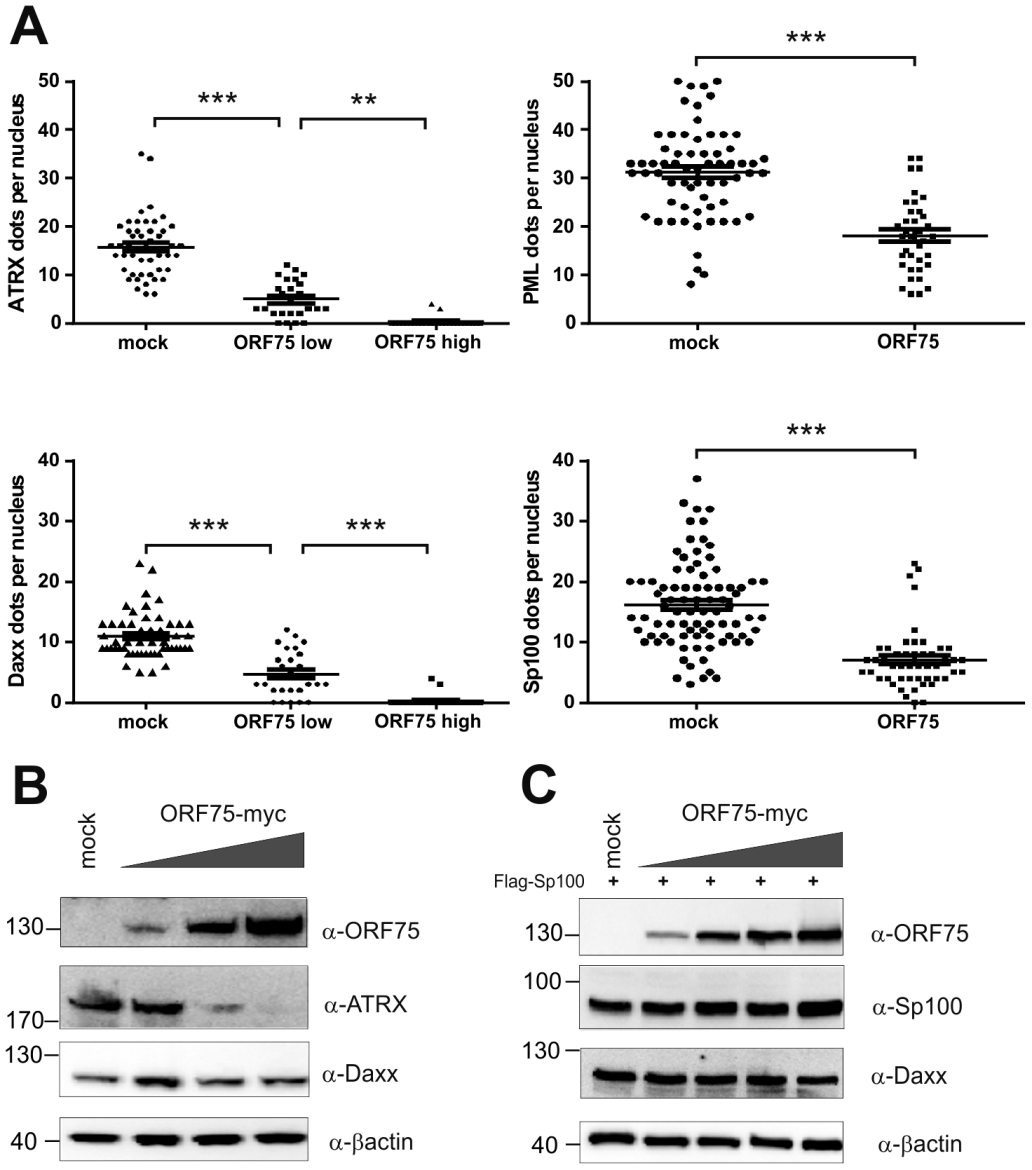
KSHV ORF75 is sufficient for antagonization of ATRX and Sp100. **A**: HFF cells were transfected with a pcDNA-orf75-myc expression construct. Cells were analyzed by immunofluorescence with a confocal microscope, and at least 50 individual cells per each box analyzed by Fiji. Statistical analyses were conducted using the unpaired t test for SP100 and PML, and one way ANOVA test for multiple comparisons for Daxx and ATRX, respectively, of GraphPad Prism 6 for Windows. **p<0.01; *** p<0.001. **B**: 293T cells were transfected with increasing amounts of ORF75 and endogenous expression of ATRX and Daxx was analyzed by immunoblot. **C**: 293T cells were cotransfected with constant levels of Sp100 and increasing amounts of ORF75 and expression of Sp100 and Daxx levels analyzed by immunoblot.

We had recently found that the lytic orf3 gene-product of the related primate rhadinovirus HVS induces the proteasomal degradation of Sp100 [16]. Thus, we speculated that the homologous ORF75 protein of KSHV may be involved in the depletion and relocalization of ND10 components. To investigate this, primary human fibroblasts were transfected with expression constructs for ORF75, and the integrity of ND10 components was visualized by multicolor immunofluorescence. The ORF75 protein shows a colocalization with the main ND10 component PML (Figure 5A and B). Moreover, compared to untransfected cells, ORF75 induced the relocalization of PML. However, we never observed a complete loss of spotted PML staining but a decrease in the number of ND10 dots per nucleus, and the remaining dots showed an increase in size (Figure 5). In addition, ORF75 induced the removal of ATRX, which is in accordance with ATRX disappearing after *de novo* KSHV-infection (Figure 5B, panels f to k and Figure 4). Furthermore, the spotted ND10-associated appearance of Daxx was lost in cells expressing ORF75 compared to untransfected cells (Figure 5A panels f,g). Quantification of ND10-dots per nucleus revealed a significant reduction in the number of PML-, Sp100-, Daxx- and ATRX-dots per cell (Figure 6A). It is worth to note, that the variation in dot number per nucleus in staining of different ND10 components is most likely related to the signal/noise ratio of used antibodies. The loss of ATRX and Daxx dots per nucleus seemed to be dependent on ORF75 levels; cells expressing high amounts of ORF75 showing both nuclear and cytoplasmic staining of ORF75 had a complete loss off ATRX and Daxx dots, whereas cells with ORF75 staining restricted to nuclei showed only reduced numbers of dots (Figure 5). Along this line, by transfection of myc-tagged ORF75 into 293T cells, we could demonstrate that ORF75 leads to a dose dependent loss of ATRX expression as detected by Western Blotting (Figure 6B). In contrast to ATRX, Daxx expression levels remained unaffected (Figures 6B+6C). Moreover, we could demonstrate that transfection of increasing amounts of ORF75-myc had no influence on Sp100 protein levels, indicating that ORF75 does not mediate a degradation of Sp100 (Figure 6C). In cells expressing high amounts of ORF75, we detected a relocalization and frequently also a complete loss of spotted Sp100 staining; PML was relocalized to more intense spots but not dispersed. This suggests that ORF75 is involved in the dispersion of both Sp100 and Daxx during lytic replication but not in Sp100 degradation (Figure 5A, panel e–h; 5B, panels b–d; g–i, Figure 6).

### KSHV ORF75 is a tegument protein and part of the virion

**Figure 7:**
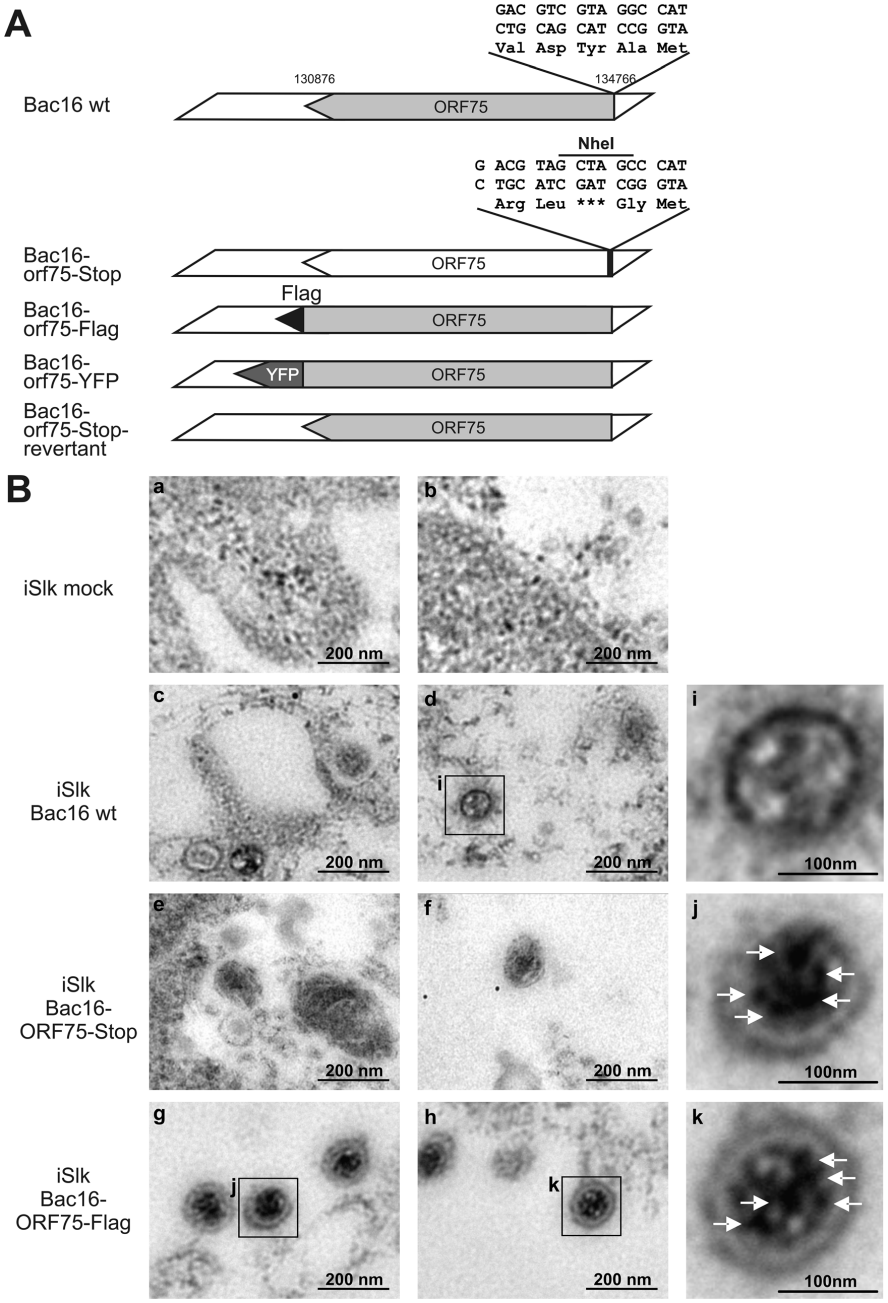
KSHV ORF75 is incorporated into the virion. **A**: Schematic representation of recombinant viruses **B**: iSLK (a, b), iSLK-Bac16 wt (c, d), iSLK-Bac16-orf75-Stop (e, f) and iSLK-Bac16-orf75Flag cells (g, h) cells were induced with sodium-butyrate and doxycyclin, fixed, embedded and subjected to immunogold staining with a Flag-specific antibody. Enlarged inserts of iSLK-Bac16 wt (i) and iSLK-Bac16-orf75Flag cells (j, k) demonstrate absence (i) or presence (j, k) of immunogold labeling in the virion particles, respectively (arrows).

The depletion of ATRX in de novo infected human fibroblasts, which are not fully permissive for KSHV, hinted at the involvement of a virion protein. We then wanted to address the question whether ORF75 is truly a tegument protein and therefore part of the virion, as it was assumed in previous studies [23], [24]. To this end, recombinant viruses were generated for further analysis of the role of KSHV ORF75 (Figure 7A). The orf75 gene is located close to the right end of the L-DNA. We generated recombinant KSHV mutants by using recombination-mediated, site-directed mutagenesis of cloned KSHV BACmid Bac16 [25]. Mutant viruses were verified using *Xho*I restriction enzyme digestion, PCR, PFGE, sequencing of recombination junctions, and Illumina sequencing of the Bac16-75-Stop-revertant and -orf75-Flag (data not shown). Pulsed field gel electrophoresis shows expected restriction patterns of wild type and deletion mutant BACs, including the full maintenance of the terminal repeat sequences (data not shown). All of these experiments proved that the recombinant genomes contained the correct deletion, with no other detectable rearrangements, and were therefore used for further analysis. iSLK cells were transfected with KSHV Bac16 or Bac16-orf75-Flag DNA, selected with hygromycin B, and lytic viral replication induced with doxycyclin and sodium-butyrate. Three days post induction, cells were harvested and Flag-tagged ORF75 was stained by immune electron microscopy (IEM) with a Flag-tag specific antibody and a secondary antibody coupled to 12 nm gold-particles. Electron microscopy imaging revealed a tegument specific gold staining of virions in iSLK-Bac16-Flag cells, which was absent in iSLK-Bac16 wt cells, indicating that the ORF75 protein is truly part of the viral particle (Figure 7B). We could not detect virus particle production by IEM in iSLK-Bac16-orf75-Stop cells after lytic viral induction despite multiple attempts (Figure 7B).

### The orf75 gene is essential for lytic replication of KSHV

**Figure 8:**
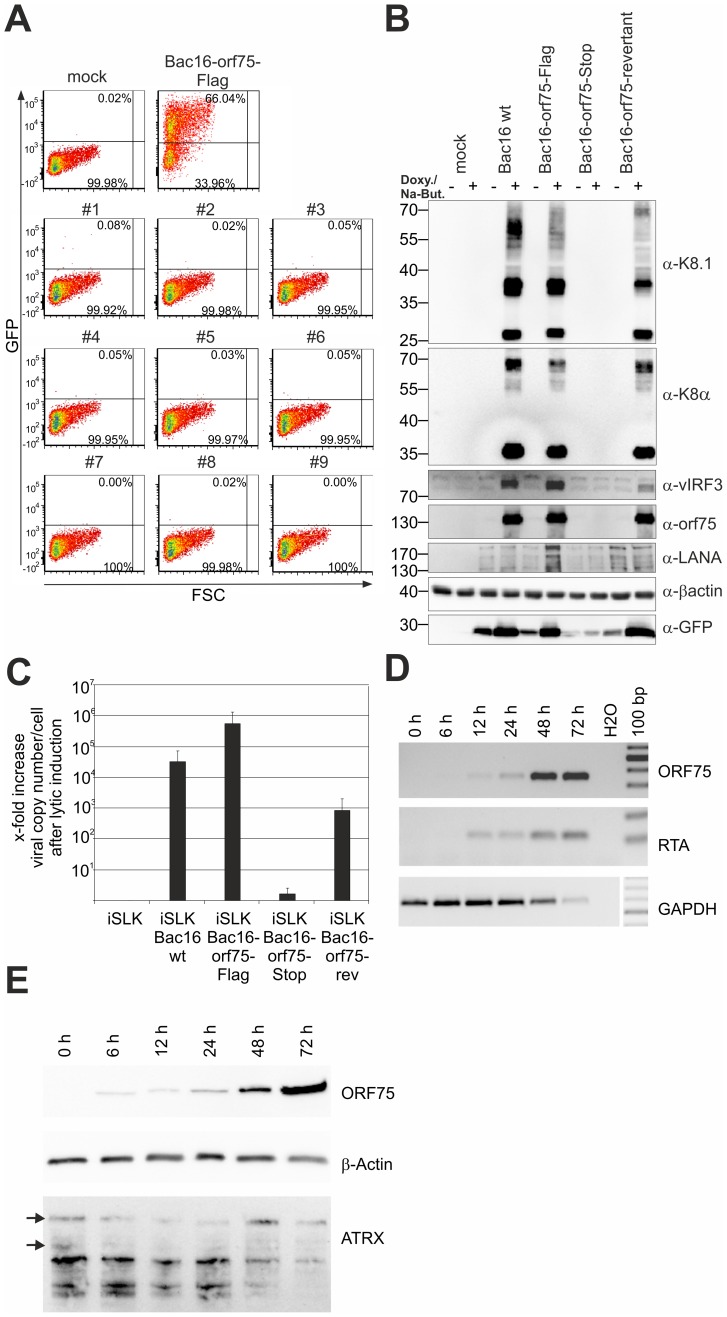
KSHV ORF75 is essential for viral replication. **A**: nine individual clones of iSLK-Bac16-orf75-Stop cells (#1–#9) were induced with sodium-butyrate and doxycyclin for 3 days, the supernatants harvested and transferred onto freshly seeded 293T cells. Two days post transfer, 293T cells were harvested and GFP expression analyzed by flow cytometry. iSLK-Bac16-orf75Flag cells served as positive control, mock infected cells as negative control. **B**: iSLK cells harboring different recombinant KSHV-BACmids were induced with sodium-butyrate and doxycyclin for 2 days, whole cell lysates prepared, and protein expression of viral genes analyzed by immunoblotting. **C**: iSLK cells harboring different recombinant BACmids were induced with sodium-butyrate and doxycyclin for 2 days, cells harvested, subjected to proteinase K digestion, and copy number of KSHV genomes relative to cellular DNA determined by quantitative real time PCR. Graphs show x-fold increase in viral copy number per cell after lytic induction and represent the mean of 3 independent experiments including standard deviation. **D**: ORF75 mRNA is already detectable early after infection of SLK cells. RT-PCR was performed with DNAse treated total RNA extracted from SLK cells newly infected with rKSHV.219 virus for the indicated times. Expression of the mRNA encoding the lytic R transactivator encoded by orf50 and of cellular GAPDH is for reference. **E**: Time course of ORF75 protein after de novo infection of SLK cells as in D; 20 µg total cellular protein per lane were resolved by SDS-PAGE and ATRX, ORF75 and beta-Actin were analyzed by immunoblot.

Recombinant viruses were reconstituted by transfection of BAC DNA into iSLK cells followed by selection with hygromycin B. Cells were treated with sodium-butyrate and doxycyclin to induce lytic virus production. We were able to reconstitute recombinant viruses carrying Flag-tagged or YFP-tagged versions of the ORF75 protein, however, despite multiple attempts we failed to reconstitute virus carrying a STOP codon at the beginning of the orf75 gene. Nine individual iSLK-Bac16-orf75-Stop clones were tested for the production of infectious virus by transfer of supernatant from induced iSLK-Bac16-orf75-Stop clones onto 293T cells; GFP-expression resulting from KSHV infection was measured by flow cytometry two days post infection. While the control showed infection rates above 50%, we could not detect infection of 293T cells using supernatant of any of the nine orf75-Stop clones (Figure 8A). Furthermore, KSHV ORF75 appears to be essential for lytic viral induction, since we could not detect lytic viral gene expression in the absence of ORF75 (Figure 8B). iSLK cells harboring different recombinant Bac16 clones were induced with doxycyclin and sodium-butyrate for 2 days and lytic gene expression determined by Western Blotting. Intriguingly, we could not detect expression of KSHV genes K8, K8.1, and v-IRF3 in iSLK-Bac16-orf75-Stop cells. In contrast, a Bac16-orf75-revertant, constructed by repairing the engineered stop-codon of the orf75-Stop mutant, showed lytic gene expression comparable to that of the wildtype, indicating that the defects observed in the orf75-Stop mutant are in fact due to a lack of orf75 expression. Further support for the essential role of ORF75 comes from knockdown experiments of ORF75 using siRNA and rescue of infectious virus from the iSLK-Bac16-orf75-Stop cells by lentiviral transduction with tet-on ORF75 (Figures S3 and S4). In addition, all cell lines showed regular expression levels of latent genes Lana/orf73 and GFP expressed from the viral genome which increased after lytic induction in all cell lines except iSLK-Bac16-orf75-Stop cells. Finally, when tested for viral DNA replication in iSLK cells by quantitative real time PCR, we could detect only a minimal increase in viral copy numbers per cell in iSLK-Bac16-orf75-Stop cells after lytic viral induction, whereas the controls showed a normal increase in copy numbers corresponding to lytic replication (Figure 8C). This might indicate that, in addition to being a tegument protein important for infectious virion production, ORF75 could have an additional role in generating a permissive environment for viral lytic DNA replication. Accordingly, orf75-mRNA can be detected as early as RTA-mRNA after infection of SLK cells (Figure 8D). In addition, ORF75 protein is present in infected cells during the entire viral replication cycle, starting from 6 h post infection up to 72 h post infection (Figure 8E).

### KSHV ORF75 colocalizes with PML in cells undergoing lytic viral replication

**Figure 9:**
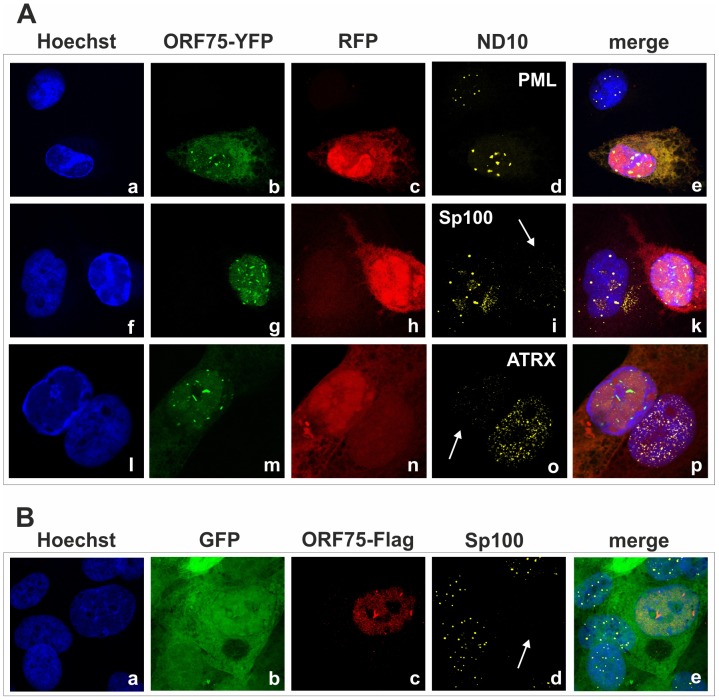
KSHV ORF75 colocalizes with ND10 in cells undergoing lytic replication. **A**: iSLK-Bac16-orf75-YFP cells were seeded onto glass slides, induced with sodium-butyrate and doxycyclin for 2 days and subjected to analysis of expression of different ND10 components by immunofluorescence. Cells carrying the Bac16-vector are depicted in red, as this backbone here includes RFP instead of GFP. **B**: iSLK-Bac16-orf75-Flag cells were seeded onto glass slides, induced for 2 days as above and subjected to analysis of expression of Sp100 by immunofluorescence. Arrows indicate induced cells expressing ORF75-YFP and ORF75-Flag respectively.

**Figure 10:**
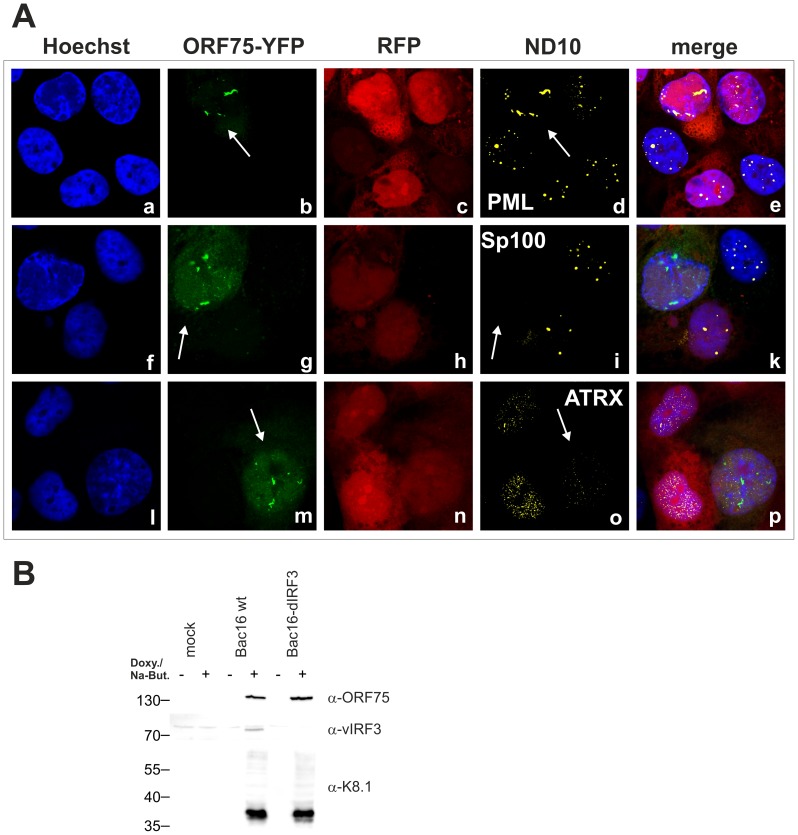
KSHV mediated depletion and relocalization of ND10 components is independent of v-IRF3. **A**: iSLK-Bac16-ΔIrf3-orf75-YFP cells were seeded onto glass slides, induced with sodium-butyrate and doxycyclin for 2 days and subjected to analysis of expression of different ND10 components by immunofluorescence. Cells carrying the Bac16-vector are depicted in red, as this backbone here includes RFP instead of GFP. Arrows indicate induced cells expressing ORF75-YFP. **B**: Expression of v-IRF3. iSLK, iSLK-Bac16 or iSLK-Bac16-ΔIrf3 cells were induced with sodium-butyrate and doxycyclin for 2 days and of expression of v-IRF3, ORF75 and lytic K8.1 protein was detected by immunoblotting. v-IRF3 is absent from iSLK cells harboring a Bac16-ΔIrf3.

In order to further clarify the role of KSHV-ORF75 during lytic replication, we used the recombinant viruses expressing ORF75 either with C-terminal Flag- or YFP-tag. After lytic induction of iSLK cells harboring the respective BACs, ORF75-YFP as well as ORF75-Flag colocalize with PML (Figure 9A and 9B). Moreover, only cells undergoing lytic replication and subsequently showing ORF75 expression exhibited a loss of both ATRX and Sp100 expression, revealing the role of ORF75 in ND10 antagonization. KSHV vIRF3/LANA2 overexpression has previously been associated with redistribution of PML, and a moderate decrease in the number of ND10 dots per cell [13]. Therefore, to separate the effects of ORF75 and vIRF3 on ND10 composition, we generated a recombinant virus expressing ORF75-YFP with additional deletion of the vIRF3 gene. After lytic induction of iSLK-Bac16-Δvirf3-orf75-YFP cells, ORF75-YFP colocalizes with PML (Figure 10). In addition, this experiment recapitulated the loss of both ATRX and Sp100 expression (Figure 10), and clearly shows that vIRF3 is not required for the ORF75-mediated alterations of ND10 composition.

### KSHV Bac16-orf75-Stop cells can be complemented by knock-down of ATRX

**Figure 11:**
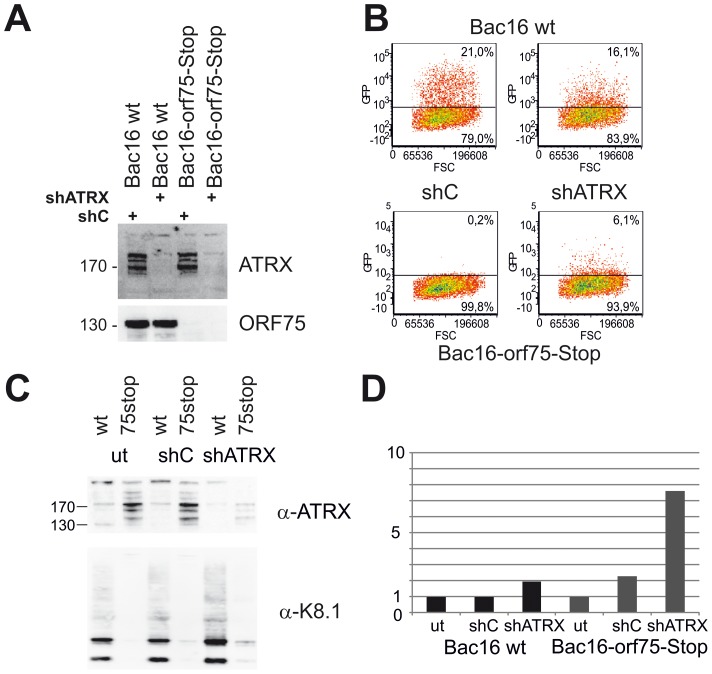
KSHV Bac16-orf75-Stop cells can be complemented by knock-down of ATRX. **A**: iSLK-Bac16 wt cells and iSLK-Bac16-orf75-Stop cells were transduced with lentiviral shRNA vectors for ATRX (shATRX) and a non-silencing control (shC). After transduction, expression of ATRX and ORF75 was analyzed by immunoblot. **B**: iSLK-Bac16 wt cells and iSLK-Bac16-orf75-Stop cells were untransduced (ut) or transduced with lentiviral shRNA vectors for ATRX and a non-silencing control, induced with doxycyclin and sodium-butyrate for 3 days and supernatants transferred onto freshly seeded SLK cells. Two days after transfer of supernatants, cells were subjected to flow-cytometric analysis of viral infection, determined by GFP expression. **C**: Viral lytic K8.1 glycoprotein expression is enhanced by ATRX knockdown. iSLK-Bac16 wt cells (wt) and iSLK-Bac16-orf75-Stop (75stop) cells were untransduced (ut) or transduced with lentiviral shRNA vectors for ATRX (shATRX) or a non-silencing control (shC) and induced as above. **D**: Quantification of K8.1 chemoluminescence signal from C using AIDA image analyzer software v4.21; signals were normalized to the respective untransduced control lane. The expression of K8.1 shows a stronger relative increase in iSLK-Bac16-orf75-Stop cells (Bac16 75stop) in the absence of ATRX compared to iSLK-Bac16 wt cells (Bac16 wt), in which ATRX is already reduced by the ORF75 protein.

We demonstrated that KSHV ORF75 mediates the depletion of ATRX and is essential for lytic viral replication. To this end, we addressed the question whether a knock-down of ATRX can re-establish lytic viral replication in the absence of ORF75. iSLK-Bac16-orf75-Stop cells were transduced with a lentiviral shRNA vector targeting ATRX (shATRX) and a respective non-silencing control vector (shC). The ATRX-specific shRNA mediates an almost complete knock-down of ATRX protein in iSLK cells (Figure 11A). After lytic induction of knock-down cells with doxycyclin and sodium-butyrate for three days and transfer of supernatant onto freshly seeded 293T cells, we could not detect infection of cells with supernatant of iSLK-Bac16-ORF75-Stop cells treated with shC (Figure 11B). However, supernatants from iSLK-Bac16-orf75-Stop cells treated with shRNA specific for ATRX contained infectious virus capable of infecting 293T cells, indicating that a viral ORF75 deletion mutant can be rescued by knock down of ATRX (Figure 11B). ATRX knockdown also leads to enhanced lytic viral gene expression. In iSLK-Bac16-orf75-Stop cells, shRNA mediated knockdown of ATRX is able to rescue expression of the lytic gene K8.1, emphasizing the role of ORF75 mediated ATRX counteraction for viral replication (Fig. 11C+D).

## Discussion

The counteraction of ND10-instituted intrinsic immunity as a critical step during the replication cycle of DNA viruses and particularly of herpesviruses has become more and more evident over the last decade. Several ND10 components, like PML, Sp100, Daxx and ATRX have been shown to be part of a cellular defense mechanism against viral infection and therefore became targets of viral effector proteins. Recently, members of the viral formylglycineamid-ribotideamidotransferase (vFGARAT) family have been added to the list of herpesviral ND10-antagonists. We demonstrated that one of the two vFGARATs of Herpesvirus *saimiri* - the ORF3 protein - leads to a selective proteasomal degradation of the ND10 component Sp100 [16]. Moreover, another vFGARAT, the BNRF1 protein of Epstein-Barr virus, has recently been shown to interact with the ND10 component Daxx, resulting in a release of the chromatin remodeling factor ATRX from ND10, and thereby facilitating viral replication [18]. One out of three viral FGARAT proteins of the murid gammaherpesvirus 68 (MHV-68), ORF75c, has been shown to fully disperse cellular ND10 complexes by inducing the proteasomal degradation of PML; it further reduced SP100 isoforms and SUMOylation, and reduced Daxx levels [17], [26]. Thus, although vFGARAT homologs BNRF1, ORF75c and ORF3 all target ND10 components, a comparative analysis of vFGARAT proteins revealed that the ORF3 FGARAT homologs, the rhadinoviral ORF75 homologs, and the lymphocryptoviral BNRF1 homologs cluster within different branches of a phylogenetic tree [16]. Apparently, viral FGARAT homologs have evolved in different ways, probably reflecting different viral survival strategies. Whereas MHV-68 is able to antagonize PML, which drastically enhances viral lytic replication, KSHV and HVS are not able to counteract PML-mediated restriction per se (Figure 1C).

It is notable in this context that the outcome of KSHV infection *in vitro* is by default latency, whereas the situation *in vivo* in humans is very difficult to assess. Therefore, it is highly remarkable that there is an increase in viral gene expression after depletion of both PML and Sp100, as shown by *de novo* GFP expression (Figure 1). According to the current model that ND10 components act as restriction factors by creating a repressive environment for viral gene-expression, this increase in GFP expression in PML- and Sp100-knock-down fibroblasts is most likely due to increased viral lytic gene expression in the absence of ND10 factors. KSHV infection leads to an initial selective depletion of ATRX and relocalization of Daxx, whereas PML and Sp100 remain unaffected (Figures 1D and 2A and B). This is in coincidence with our data of KSHV infection of ATRX knock-down cells: since ATRX is efficiently antagonized by the virus, there is no further enhancement of KSHV infection after ATRX depletion (Figure 1). We could further observe a modest increase in GFP-expression after Daxx depletion, indicating that Daxx can only be partially antagonized by KSHV.

Alpha- and beta-herpesviruses, like herpes simplex virus and human cytomegalovirus, have evolved very efficient counter-mechanisms that dissolve ND10, while primate gammaherpesviruses are lacking the full ability to counteract a central part of the ND10 intrinsic immunity. Therefore, there is good evidence to support the hypothesis that ND10 components like PML may contribute to the efficient establishment of latent infection in target cells, leading to the accumulation of secondary mutations over time and ultimately resulting in oncoprotein mediated cell transformation, as it is the case for most gamma-herpesviruses. On the other side, lytic disease and lytic reactivation is the observed outcome for most alpha- and beta-herpesviruses that can overcome ND10 restriction.

KSHV encodes a single vFGARAT homolog, the ORF75 protein. ORF75 has been described as a tegument protein, due to its homology to other vFGARATs and its presence in preparations of purified virions [23], [24]. We could confirm Flag-tagged ORF75 within KSHV virions by immune electron microscopy (Figure 7B). In addition, in a genome-wide screen for NF-kB activation by KSHV proteins, ORF75 has been shown to activate the NF-kB signaling pathway [19]. The role of ORF75 during lytic viral replication had not been assessed so far. Here we could demonstrate that the ORF75 protein of KSHV is involved in the antagonization of ND10-mediated immunity. Isolated expression of ORF75 in primary human cells led to alterations in ND10 composition reminiscent of the situation during lytic viral replication (Figures 4, 5 and 6): i) relocalization of ND10 components including PML; ii) depletion of ATRX; iii) relocalization and dispersion of Sp100; iv) dispersion of Daxx. Using quantification of immunofluorescent images and immunoblotting we could show that ORF75 acts in a dose dependent manner on ND10 components Daxx and ATRX (Figure 6). In addition we could also observe a strong colocalization of ORF75 and PML. These findings could also be confirmed during lytic viral replication, using either Flag-tagged or YFP-tagged ORF75 protein expressed by recombinant viruses (Figures 9 and 10). Cells undergoing lytic replication showed a relocalization of PML, a colocalization of ORF75-YFP and PML, exclusion of Sp100 and Daxx from ND10, and loss of ATRX protein expression; this further corroborates the role of ORF75 in ND10 antagonization.

For the generation of infectious viruses from recombinant KSHV genomes, we used iSLK cells transfected with BAC DNA. Although it has recently been demonstrated that iSLK cells are not of endothelial origin as it has been assumed previously [22], this cell line can be used for efficient virus production. According to our experience, the internal repeat and multiple TR sequences present in KSHV mandate extra caution. In contrast to other BAC-cloned herpesvirus genomes, which seem to be rather stable during homologous recombination in *E. coli*, we frequently observed shortening of terminal repeat sequences from Bac16 recombinants. Since this cannot be detected by conventional gel electrophoresis and DNA sequencing, it is notable that we routinely verify KSHV BAC16 recombinants by pulsed field gel electrophoresis (PFGE) as well as Illumina deep sequencing of final constructs. It would have been of great interest to see whether the ablation of ORF75 expression affects the antagonization of ND10 components during infection and lytic replication. Intriguingly, we were unable to reconstitute infectious virus and could detect neither lytic gene expression nor viral DNA replication in the absence of ORF75. The expression of latent genes Lana/orf73 and GFP was not affected in unstimulated iSLK cells. Thus, to our own surprise, ORF75 seems to be essential for lytic replication at multiple levels. We could show for HVS that individual deletion of vFGARATs ORF3 and ORF75, respectively, leads to an impaired viral replication, but did not entirely abrogate viral replication [16]. However, for HVS, we were so far not able to reconstitute a virus with deletion of both ORF3 and ORF75, hinting at the important role of at least one vFGARAT in the genome of a rhadinovirus (Full and Ensser, unpublished data). In this context, it would also be of great interest to elucidate the role of ORF75 of rhesus rhadinovirus (RRV), the closest known relative of KSHV, during lytic replication.

The loss of virus production in the absence of ORF75 hints at multiple functions of the protein within the viral life cycle, as it has also been shown for ORF75c of MHV68 [26]. Since ORF75 is a tegument protein (Figure 7B), it may well be that it is an integral part of the virion and necessary for assembly of viral particles. However, the loss of an important structural component of the virion would not explain the complete absence of lytic gene expression and viral replication. In ORF75c-deleted MHV68 viral capsids are still made and accumulate, while no particles are apparent in orf75-stop KSHV (Figure 7B). It is thus remarkable that we cannot detect lytic gene expression despite overexpression of RTA in induced iSLK cells (Figure 8B). It is also unlikely that the loss of K13/vFLIP-mediated NF-kB (co)activation by ORF75 leads to this effect, as the KSHV genome comprises multiple potent activators of NF-kB signaling [19]. Our data hint at a role of ORF75 in the initiation of lytic gene expression. As a prerequisite, ORF75 protein must be expressed during latency or at least early during initiation of the lytic cycle. This is indeed the case (Figure 8D+E): after infection, ORF75 is released from the viral tegument into the cell. As soon as immediate early gene expression starts, de novo ORF75 protein is made in small amounts and at later timepoints in increasing amounts until new viral particles are released form the cell. Thus ORF75 protein is present for the entire viral replication cycle. The orf75 gene is situated within a region at the right end of the KSHV genome that is transcribed during latency, and genome-wide analysis of epigenetic modifications of latent KSHV genomes revealed histone modification patterns in the putative orf75 promoter region which reflect actively transcribed chromatin [27], [28]. Therefore, it would be very interesting, but beyond the scope of this article, to test for potential epigenetic mechanisms underlying the loss of viral DNA replication in the absence of ORF75, and the role of ORF75 during latency of KSHV.

Concerning the role of ORF75 during lytic replication, we could clearly show that ORF75 is essential for viral replication. Moreover the dispersion of ATRX by ORF75 seems to be crucial for lytic replication of KSHV; a KSHV-orf75 deletion mutant that is not capable of lytic viral replication can at least be partially rescued by knock-down of ATRX (Figure 11), clearly emphasizing the role of ORF75 mediated ATRX relocation. The fate of ATRX after ORF75 mediated removal from ND10 remains elusive. Proteasomal degradation of ATRX can be excluded (Figure 3A+B). Although difficult to prove, we hypothesize that ATRX might be translocated to a highly insoluble nuclear fraction, and therefore can no longer exert its antiviral function.

KSHV infection leads to a dispersion of the ND10 component ATRX (Figures 1D and 2). We could not detect a degradation of Daxx, although KSHV infection also led to a loss of spotted Daxx localization (Figures 1D, 2, 3). Apparently, Daxx is also dispersed from ND10. Whether the Daxx dispersion is a direct or indirect effect of KSHV-induced loss of ATRX could not be demonstrated. Using co-immunprecipitation experiments, we could not detect a direct interaction of ORF75 with Daxx as it has been shown for BNRF1 of EBV [18]. This is also in accordance with findings by Tsai et al [18], indicating that EBV- and KSHV-FGARATs target the same proteins but have evolved different mechanisms in the antagonization of ND10 components. Isolated expression of ORF75 after transfection led to alterations in ND10 composition similar to observations after lytic replication. In order to separate the functions of vIRF3 and ORF75, we constructed a recombinant virus harboring a deletion of the vIRF3 gene in combination with either YFP-tagged ORF75 (Figure 10) or Flag-tagged ORF75 protein (data not shown). It has been shown that vIRF3 is SUMOylated, and that overexpression of vIRF3 leads to a moderate decrease in ND10 foci per cell [13], [29]. However, the phenotype of vIRF3-deletion mutants compared to wildtype virus with respect to ND10 was indistinguishable. Since it is known, that ND10 components can be induced by IFN, the decrease in ND10 dots per cell may also be a result of IFN antagonization by vIRF3 [30]–[32].

In summary, our results clearly revealed that ORF75 is essential for viral replication. Thus, ORF75 could be a new and specific target for antiviral therapy, since inhibition of ORF75 function should abrogate viral lytic replication. This is of particular interest also for therapy of KSHV tumors, since it is known that lytic replication of KSHV is of particular importance for KS tumorigenesis *in vivo*. KS tumors regularly show lytic viral replication at low levels [33], [34], and a clinical trial demonstrated that the development of new KS lesions in patients can be blocked by administration of ganciclovir, an inhibitor of the viral DNA polymerase which is required for lytic viral replication [35], [36].

## Materials and Methods

### Cells and viruses

Primary human foreskin fibroblasts (HFF), were prepared from human foreskin-tissue as described previously [37] and HFF with a small interfering RNA-mediated knockdown of PML (siPML cells), Daxx (siDaxx cells), Sp100 (siSp100 cells), ATRX (siATRX cells) and the respective control cells (siC cells) were cultured in Dulbecco's minimal essential medium (DMEM, LifeTechnologies, Germany) supplemented with 10% fetal calf serum and 5 µg/ml puromycin [38]–[40]. HeLa cells and HEK 293T cells were maintained in Eagle's minimal essential medium (LifeTechnologies) supplemented with 10% fetal calf serum. iSLK cells were maintained in DMEM supplemented with 10% fetal calf serum, 2.5 µg/ml puromycin and 250 µg/ml G418. KSHV from BAC-DNA was obtained by reconstitution of infectious viruses after transfection of BAC16 DNA into iSLK cells. iSLK cells were transfected in 6-wells with 5 µg BACmid DNA with 9 µl ExtremeGene (Roche) according to manufacturer's instructions, and selected with 200 µg/ml hygromycin B until all cells were GFP positive.

Lytic replication of KSHV was induced in iSLK harboring KSHV Bac16-DNA cells by plating cells in the absence of antibiotics and adding 900 µM sodium-butyrate and 1 µg/ml doxycyclin. UV inactivation was done by application of 120.000 microjoules/cm^2^ using the Stratalinker UV Crosslinker 1800 (Stratagene, Amsterdam, the Netherlands). Supernatants containing infectious virus were harvested 3 d post induction and stocks of wildtype and recombinant viruses were stored at −80°C in aliquots. Viral replication in induced iSLK cells was monitored by quantitative PCR of the KSHV orf26 locus and compared to cellular ccr5 DNA.

### BAC mutagenesis and plasmids

The KSHV Bac16 clone was used for recombination-mediated genetic engineering of recombinants by a two-step, markerless λ-red-mediated recombination strategy using the kanamycin gene as a first selection marker [41]. Linear fragments for homologous recombination were generated by PCR using Phusion High Fidelity DNA polymerase (Finnzymes); *Dpn*I was added to digest the methylated plasmid template, and the amplification product was purified from an agarose gel with the Nucleobond Gel Extraction Kit (Macherey&Nagel, Düren, Germany). Primers that were used to generate linear fragments for the manipulations were purchased as Ultramers™ from Integrated DNA Technologies.

For homologous recombination, the PCR fragment was then transformed into *Escherichia coli* strain GS1783 (gift of G. Smith, NW University, USA) [42] harboring BAC16, and Red recombination was performed as described earlier [41], [43]. Cells were then plated on agar plates containing 15 µg/ml kanamycin (first recombination) or 15 µg/ml chloramphenicol and 1% arabinose (second recombination) and incubated at 32°C for 1–2 days. Bacterial colonies growing on these plates were further analyzed. Reconstitution of recombinant KSHV using purified BAC DNA was performed in iSLK cells as described above.

### Viral nucleic acid isolation and analysis

BAC DNA was isolated by standard alkaline lysis from 5 ml liquid cultures. Subsequently, the integrity of BACmid DNA was analyzed by digestions with restriction enzyme *Xho*I and separation in 0.8% PFGE agarose (Bio-Rad) gels and 0.5×TBE buffer by pulse field gel electrophoresis at 6 V/cm, 120 degree field angle, switch time linearly ramped from 1 s to 5 s over 16 h (CHEF DR III, Bio-Rad). For characterization of insertion or deletion of the *aphaI* selection marker, recombinant BACmids were analyzed by PCR, and the recombined regions within the BAC DNA were controlled by sequence analysis (ABI 3130XL genetic analyzer, Weiterstadt, Germany) in order to confirm the correct deletion sequences and to exclude accidental mutations. Furthermore, the integrity of the complete viral genome of representative Bac16 recombinants was confirmed by next-generation sequencing using Nextera DNA Sample Preparation system and the MiSeq Reagent Kit, 300 Cycles on the Illumina MiSeq system. NGS data were analyzed by Genome Workbench 5 (CLCbio, Aarhus, DK).

### Antibodies

Endogenous PML was detected with mouse monoclonal antibody PG-M3 (IgG1, Santa Cruz Biotechnology (SCBT) or 5E10 (gift of R. van Driel, University of Amsterdam, The Netherlands). Human Sp100 was detected with a rabbit polyclonal antiserum (ProteinTech Group, USA) or a mouse polyclonal antibody (MaxPAP, Abnova, USA). A rabbit polyclonal antibody from Sigma-Aldrich (Germany) or the mouse monoclonal antibody MCA2143 (IgG1, Serotec, Germany) were used for detection of hDaxx. ATRX protein expression was detected with mouse monoclonal antibody clone D-5 (IgG2a, SCBT). Rabbit polyclonal antibody for detection of beta-actin, was purchased from Sigma-Aldrich, GFP-specific rabbit polyclonal antibody was purchased from Genescript (Piscataway, NJ). Epitope-tagged proteins were either detected using the anti-FLAG tag antibody M2 (Sigma-Aldrich, Germany) or the anti-myc tag antibodies clone 9E10 (IgG1, Sigma-Aldrich) or clone 9B11 (IgG2a, Cell Signaling Technologies, Germany). Horseradish-peroxidase-conjugated anti-mouse and anti-rabbit secondary antibodies for Western blot analysis were obtained from Dianova (Hamburg, Germany), while Alexa Fluor 488-, Alexa Fluor 555-, and Alexa Fluor 647-conjugated secondary antibodies for indirect immunofluorescence experiments were purchased from LifeTechnologies (Germany). For detection of KSHV proteins, KSHV-antibodies to K8 (clone 8C12G10G1, SCBT, USA), K8.1 (clone 4A4, SCBT, USA), vIRF3 (rat monoclonal, kindly provided by Frank Neipel) and ORF75 KSHV (polyclonal rabbit serum, Genescript) were used.

### Immunofluorescence analysis

For immunofluorescence analysis, iSLK cells or HFF cells were plated onto coverslips. Lytic replication of KSHV in iSLK cells was induced as described above.

Cycloheximide was used at 10 µg/ml, MG132 at 10 µM. At indicated timepoints post induction, infection or transfection, cells were washed three times with phosphate-buffered saline (PBS), followed by fixation with 4%paraformaldehyde for 10 min at room temperature. Then, cells were permeabilized with PBS-0.2% Triton X-100, 5% FCS for 1 hour, followed by incubation with the respective primary or secondary antibodies for 30 min at 37°C. Finally, the cells were mounted by using Mowiol mounting medium (Fluka, Germany) plus 4′,6-diamidino-2-phenylindole (DAPI; Vector Laboratories, USA) or Hoechst (Sigma-Aldrich, Germany). The samples were examined by using a Leica TCS SP5 confocal microscope, with 405 nm, 488 nm, 543 nm or 633 nm laser lines, scanning each channel separately under image capture conditions that eliminated channel overlap. The Fiji distribution of ImageJ was used to count nuclear dots in ORF75 expressing human fibroblasts [44]. Briefly, nuclei were identified by watershedding and the analyze particles function in the DAPI image, then defined as ROI; after setting an appropriate noise tolerance, single point maxima in the channel corresponding to ND10 proteins were then counted within each nuclear ROI and results copied to MS Excel. Statistical analyses were conducted using the unpaired t-test for SP100 and PML, and one way ANOVA test for multiple comparisons for Daxx and ATRX, respectively (GraphPad Prism 6 for Windows, GraphPad Software, USA).

### Western blot analysis

Extracts from infected cells were prepared either directly in a sodium dodecyl sulfate-polyacrylamide gel electrophoresis (SDS-PAGE) loading buffer or in a RIPA-lysis buffer as described previously, separated either on sodium dodecyl sulfate-containing 8%, 10% 12% polyacrylamide gels or 4–12% bis-tris gradient gels (Life Technologies, Germany), and transferred to polyvinylidene fluoride membranes (Millipore). Enhanced Chemi-luminescence was detected according to the manufacturer's protocol (GE Healthcare).

### Flow cytometry

HFF cells or 293T cells were detached using PBS supplemented with 0.1% EDTA, pH 7.4, spun down (300×g, 5 min), supernatant removed and then fixed in PBS supplemented with 2% paraformaldehyde for 30 min. GFP expression of cells was analyzed on a LSRII flow cytometer (BD Biosciences) and data were analyzed with FCS Express 3 software (De Novo Software, Canada). For cell cycle analysis, cells were harvested and fixed in 80% ethanol at least over night. Staining after RNase treatment (50 µg/ml) was done with propidium iodide (20 µg/ml) and analyzed on a BD LSR2 flow cytometer. Cell cycle data were modeled with ModFitLT 3.3 for Windows (Verity Software House, Topsham, ME).

### Immunoelectronmicroscopy

Induced iSLK cells were harvested in Dulbeco's modified Eagle's medium (DMEM) 48 h post induction. Cells were fixed with DMEM containing 4% paraformaldehyde, dehydrated at −20°C and embedded in LR-White at −20°C. Polymerization was induced by heat for 2 days at 60°C prior to sectioning using an ultramicrotome (Leica, Wetzlar, Germany). The sections were transferred to nickel grids and immunostaining was performed with anti-FLAG antibody (clone M2, Sigma-Aldrich) and secondary anti-mouse antibody conjugated with 12 nm gold (Dianova). The grids were stained for 10 min with 1% (w/v) uranyl acetate in 40% Ethanol followed by lead citrate staining for additional 10 min prior to analysis by electron microscopy. Specimens were observed using a TecnaiTM G2 Scanning transmission electron microscope (FEI Company, Eindhoven, The Netherlands) operated at 120 kV.

## Supporting Information

Figure S1 **ND10 protein expression after gene specific shRNA knockdown.** Human fibroblasts that were transduced with retroviral shRNA specific for PML (all isoforms), SP100, Daxx, ATRX were analyzed by immunoblotting using monoclonal PML (clone 5E10) or polyclonal Daxx and ATRX (SCBT) antibodies.
(TIF)

Figure S2 **Expression of ORF75-Flag and ORF75.** Exposures of immunoblots detected with ORF75 specific antiserum against an aminoterminal peptide (right), and the anti-FLAG-M2 monoclonal antibody directed at the carboxyterminal FLAG epitope (left); this demonstrates the expression of the full length ORF75 and the absence of major alternative, co-terminal gene products translated in frame from ORF75.
(TIF)

Figure S3 **Lentiviral ORF75 rescues infectious virus production from iSLK-Bac16-orf75-Stop cells.** iSLK-Bac16-orf75-Stop cells were modified by lentiviral transduction with tet-on ORF75 or empty vector construct, respectively, and lytic replication was induced with tetracycline and sodium-butyrate; after 3 days, culture supernatants transferred to empty SLK cells. 2 days post supernatant transfer, cells were subjected to flow cytometric analysis of viral infection, determined by GFP expression.
(TIF)

Figure S4 **Knockdown of ORF75 specifically reduces infectious virus production.** iSLK cells Bac16 were transfected with siRNAs specific for ORF75 (si75/1, si75/2) or controls (siC, siEGFP, siC/TYE). Lytic replication was induced with tetracycline and sodium-butyrate; after 3 days, cells were harvested for western blotting, and culture supernatants were transferred to empty SLK cells. 2 days post supernatant transfer, cells were subjected to flow-cytometric analysis of viral infection, determined by GFP expression. **A**: Western blot demonstrating successful knockdown of ORF75. **B**: Knockdown of ORF75 by si75 strongly reduces infectious virus in the supernatant of induced iSLK cells carrying KSHV Bac16.
(TIF)

Figure S5 **Diffuse localization of ATRX after knockdown of Daxx.** HFF cells carrying retroviral knockdown shRNA vectors targeting Daxx or PML were immunostained with respective antibodies. ND10 accumulation as shown by colocalization (c) with PML (a) of ATRX (b) is lost in Daxx-kd cells (e,f) while PML (d) remains in ND-10 structures. In contrast, knockdown of PML (g–i) results in dispersal of ND10 and seemingly a partial colocalization (i) of Daxx (g) and ATRX (h) into smaller structures; most of Daxx and ATRX proteins are not colocalized.
(TIF)

Figure S6 **ATRX remains disperse in infected shDaxx cells.** SLK cells mock treated (a–d), carrying knockdown shRNA vectors shC (e–h) or shDaxx (i–l) were infected by rKSHV.219 >(e–l) and immunostained with respective antibodies. The ND10 structure is detected by SP100 (for compatibility of antibody and secondary reagents). ATRX (g, arrow) is lost in infected shC (f, arrow) and seemingly also reduced in a Daxx-kd cell (j,k arrow) while Sp100 (h,l) remains in ND-10 structures. Localization of ATRX remains disperse after infection in shDaxx cells.
(TIF)

Figure S7 **Disappearance of ATRX is dependent on virus amount.** Equal numbers of SLK cells were seeded in 25 cm^2^ flasks; cells were infected with rKSHV.219 virus stock at the indicated dilutions or cultured, uninfected for 18 h; cells were harvested and expression of Actin, GFP, and ATRX was analyzed by immunoblotting.
(TIF)

Figure S8 **Infection by KSHV does not lead to cell cycle arrest in SLK cells.** Equal numbers of Slk cells were seeded in 25 cm^2^ flasks; cells were infected with rKSHV.R219 or cultured uninfected. At the indicated time, cells were harvested and fixed in 80% ethanol. Flow cytometric cell cycle analysis was done after RNase treatment (50 µg/ml) and propidium iodide (20 µg/ml)staining on a BD LSR2. Single cells were selected by gating for FSC-area vs FSC-width and PI-area vs PI-with. Relative proportions of cells in G1, S or G2 were modeled with ModFitLT 3.3 for Windows (Verity Software House, Topsham, ME).
(TIF)
