# Supplementary figures and images for: Kaposi's Sarcoma Associated Herpesvirus Tegument Protein ORF75 Is Essential for Viral Lytic Replication and Plays a Critical Role in the Antagonization of ND10-Instituted Intrinsic Immunity
Source: PLoS Pathog. 2014 Jan 16;10(1):e1003863. doi: 10.1371/journal.ppat.1003863 (PMC3894210; doi:10.1371/journal.ppat.1003863)

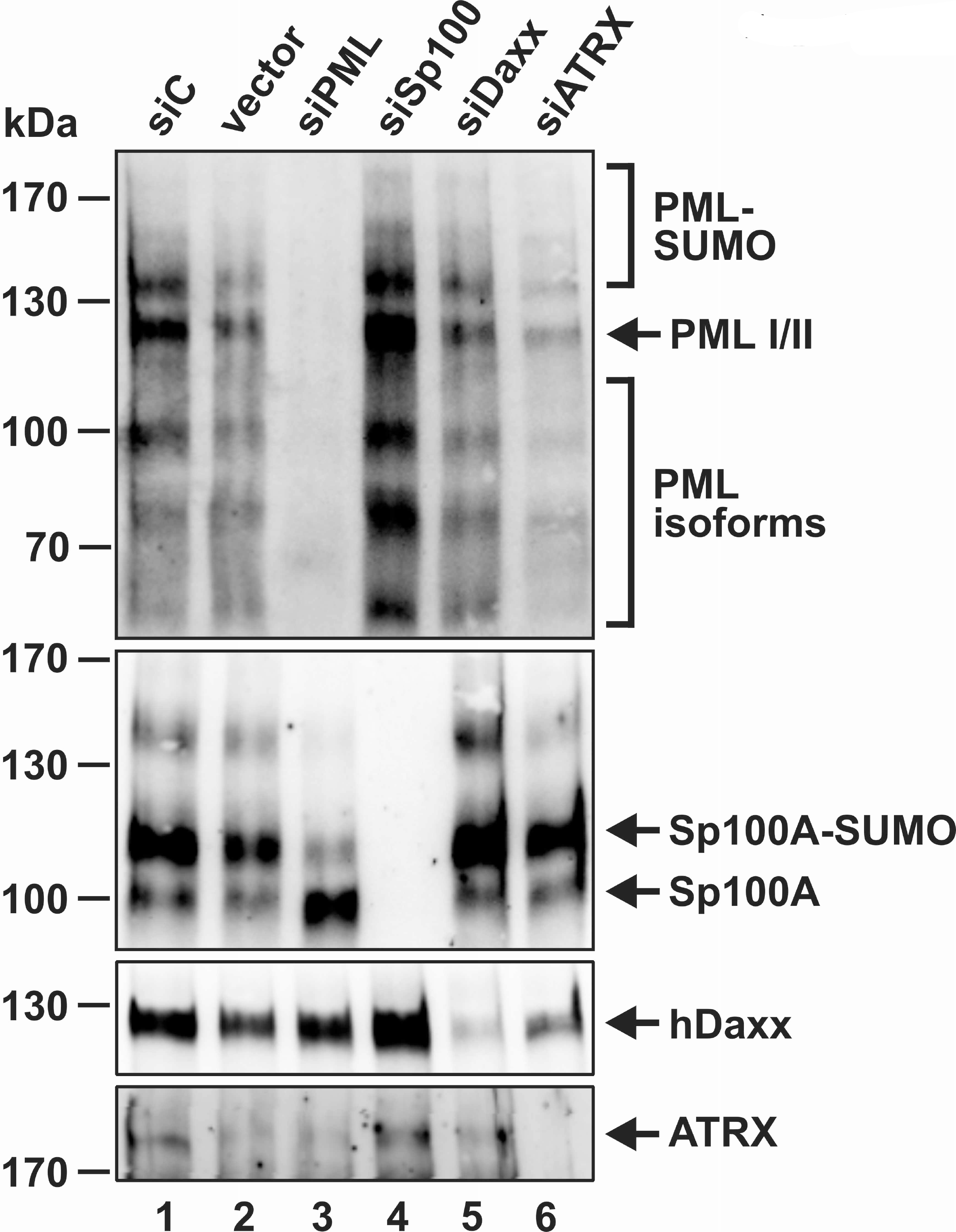

Supplement: Figure S1 — ND10 protein expression after gene specific shRNA knockdown. Human fibroblasts that were transduced with retroviral shRNA specific for PML (all isoforms), SP100, Daxx, ATRX were analyzed by immunoblotting using monoclonal PML (clone 5E10) or polyclonal Daxx and ATRX (SCBT) antibodies. (TIF) [file ppat.1003863.s001.tif]

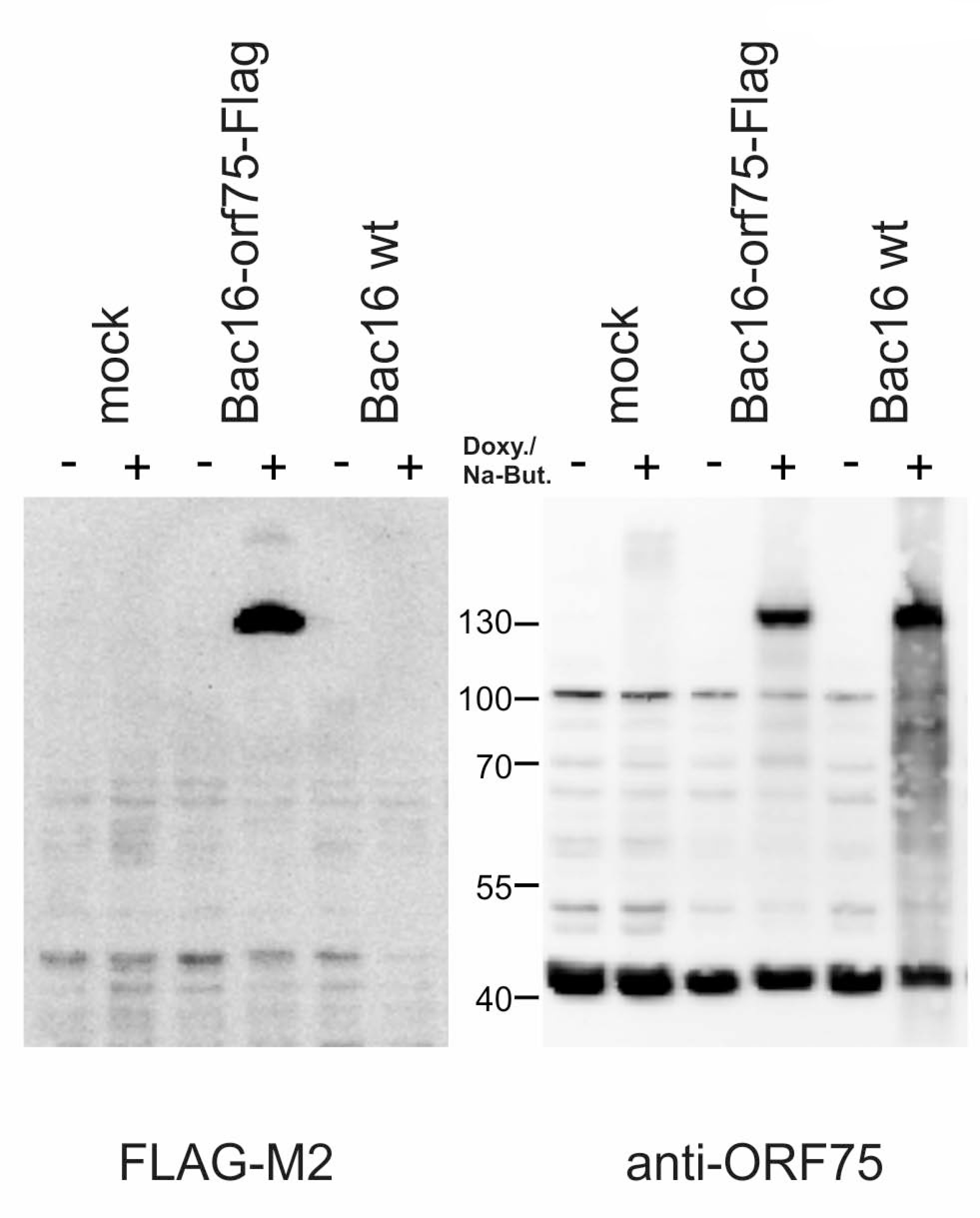

Supplement: Figure S2 — Expression of ORF75-Flag and ORF75. Exposures of immunoblots detected with ORF75 specific antiserum against an aminoterminal peptide (right), and the anti-FLAG-M2 monoclonal antibody directed at the carboxyterminal FLAG epitope (left); this demonstrates the expression of the full length ORF75 and the absence of major alternative, co-terminal gene products translated in frame from ORF75. (TIF) [file ppat.1003863.s002.tif]

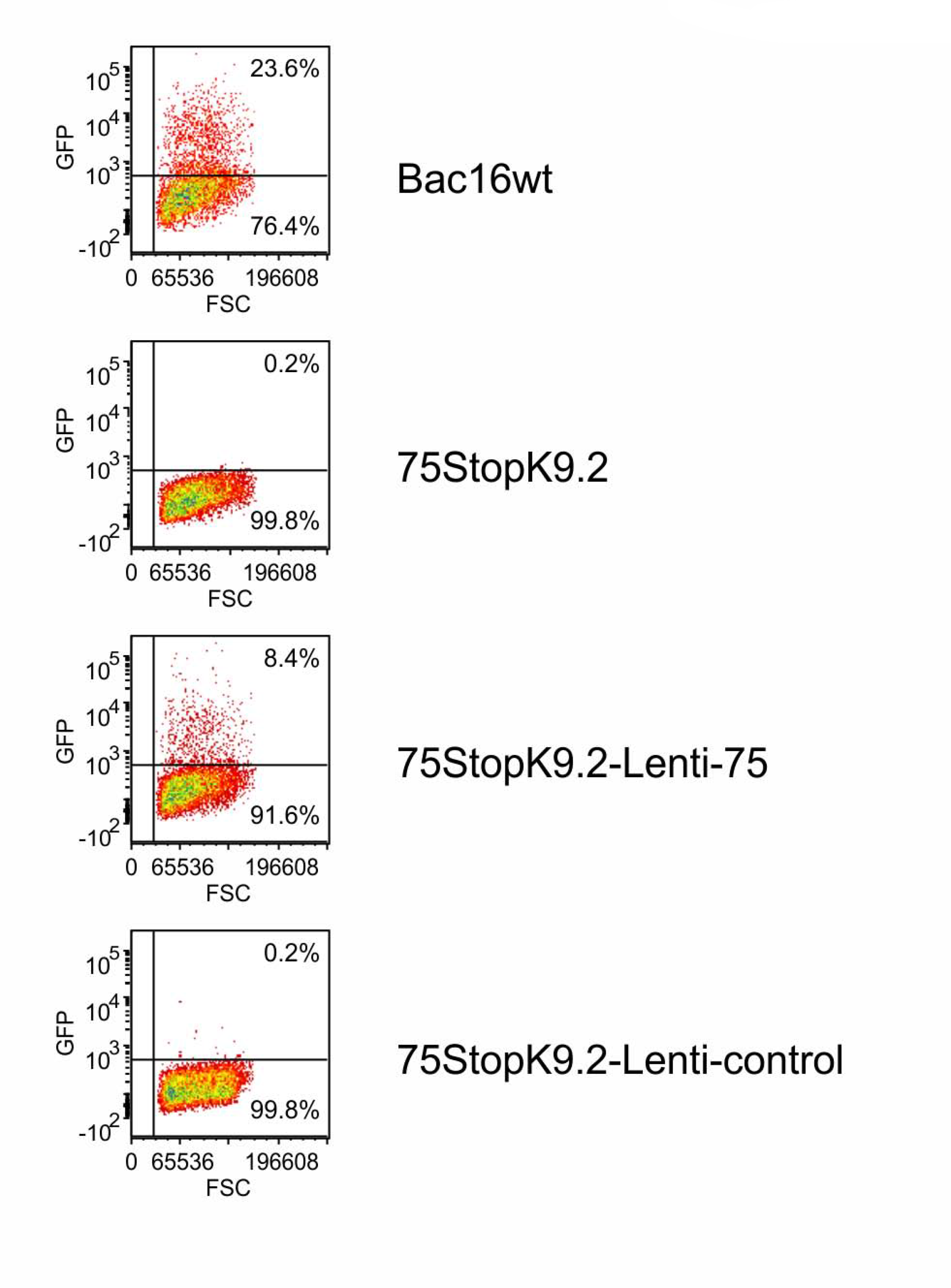

Supplement: Figure S3 — Lentiviral ORF75 rescues infectious virus production from iSLK-Bac16-orf75-Stop cells. iSLK-Bac16-orf75-Stop cells were modified by lentiviral transduction with tet-on ORF75 or empty vector construct, respectively, and lytic replication was induced with tetracycline and sodium-butyrate; after 3 days, culture supernatants transferred to empty SLK cells. 2 days post supernatant transfer, cells were subjected to flow cytometric analysis of viral infection, determined by GFP expression. (TIF) [file ppat.1003863.s003.tif]

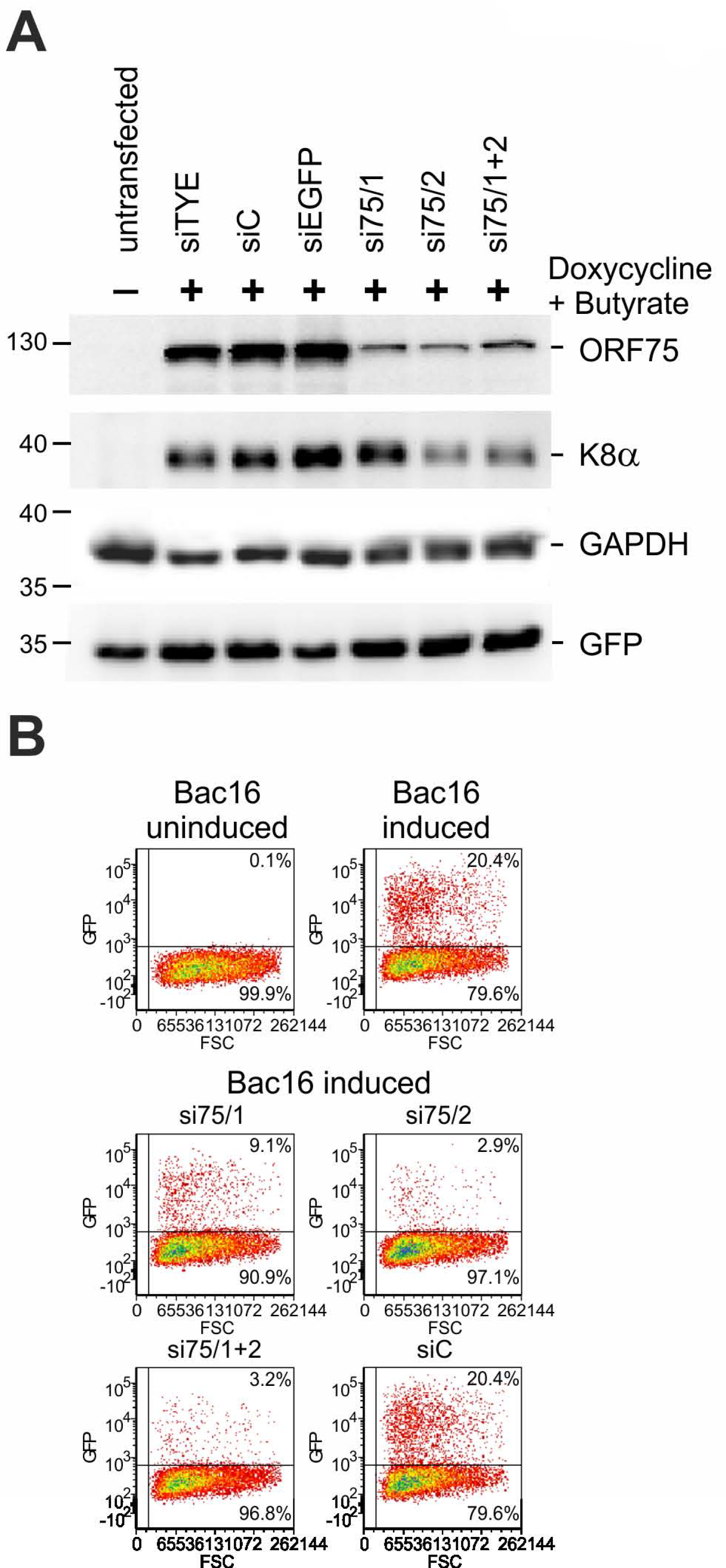

Supplement: Figure S4 — Knockdown of ORF75 specifically reduces infectious virus production. iSLK cells Bac16 were transfected with siRNAs specific for ORF75 (si75/1, si75/2) or controls (siC, siEGFP, siC/TYE). Lytic replication was induced with tetracycline and sodium-butyrate; after 3 days, cells were harvested for western blotting, and culture supernatants were transferred to empty SLK cells. 2 days post supernatant transfer, cells were subjected to flow-cytometric analysis of viral infection, determined by GFP expression. A: Western blot demonstrating successful knockdown of ORF75. B: Knockdown of ORF75 by si75 strongly reduces infectious virus in the supernatant of induced iSLK cells carrying KSHV Bac16. (TIF) [file ppat.1003863.s004.tif]

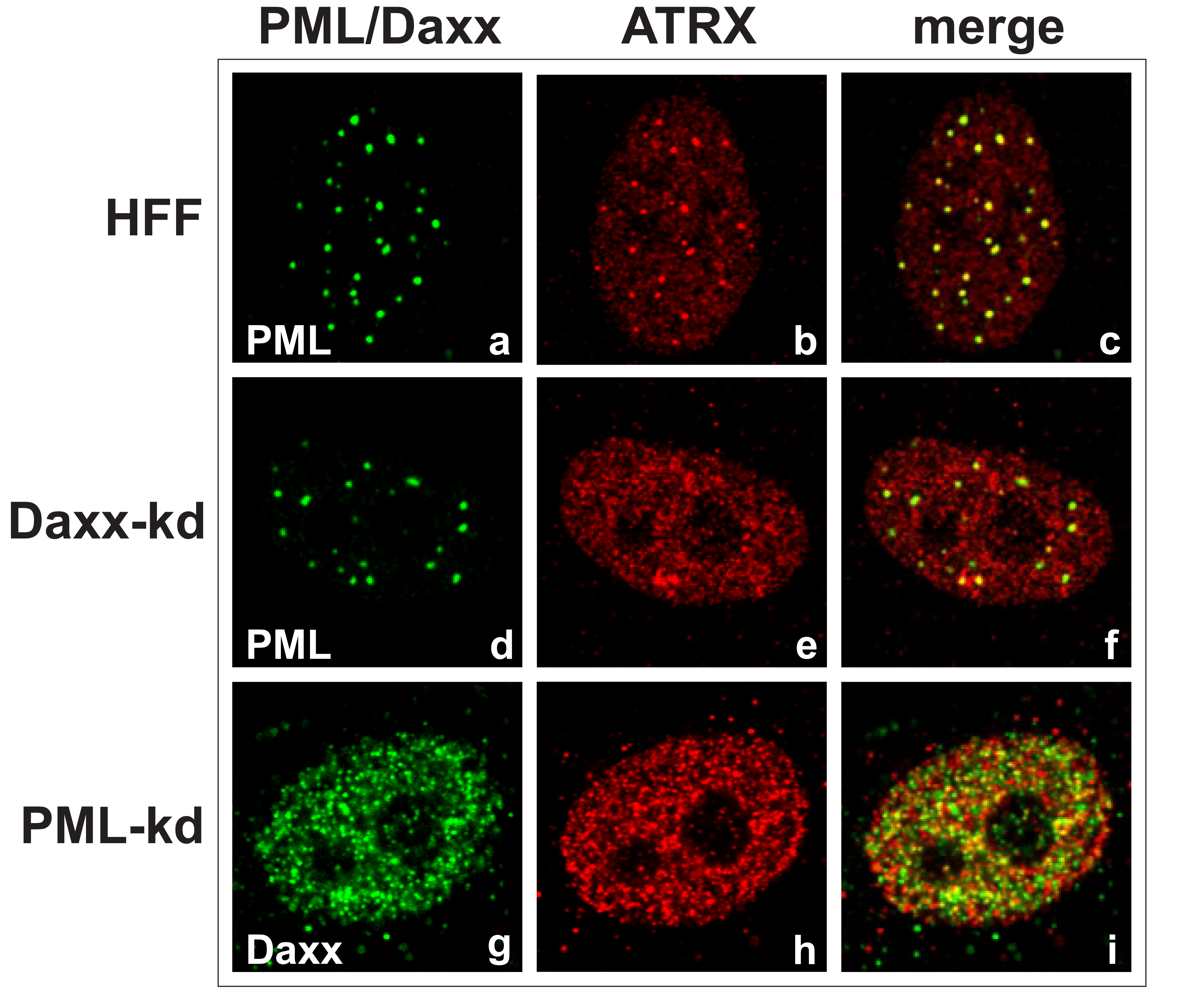

Supplement: Figure S5 — Diffuse localization of ATRX after knockdown of Daxx. HFF cells carrying retroviral knockdown shRNA vectors targeting Daxx or PML were immunostained with respective antibodies. ND10 accumulation as shown by colocalization (c) with PML (a) of ATRX (b) is lost in Daxx-kd cells (e,f) while PML (d) remains in ND-10 structures. In contrast, knockdown of PML (g–i) results in dispersal of ND10 and seemingly a partial colocalization (i) of Daxx (g) and ATRX (h) into smaller structures; most of Daxx and ATRX proteins are not colocalized. (TIF) [file ppat.1003863.s005.tif]

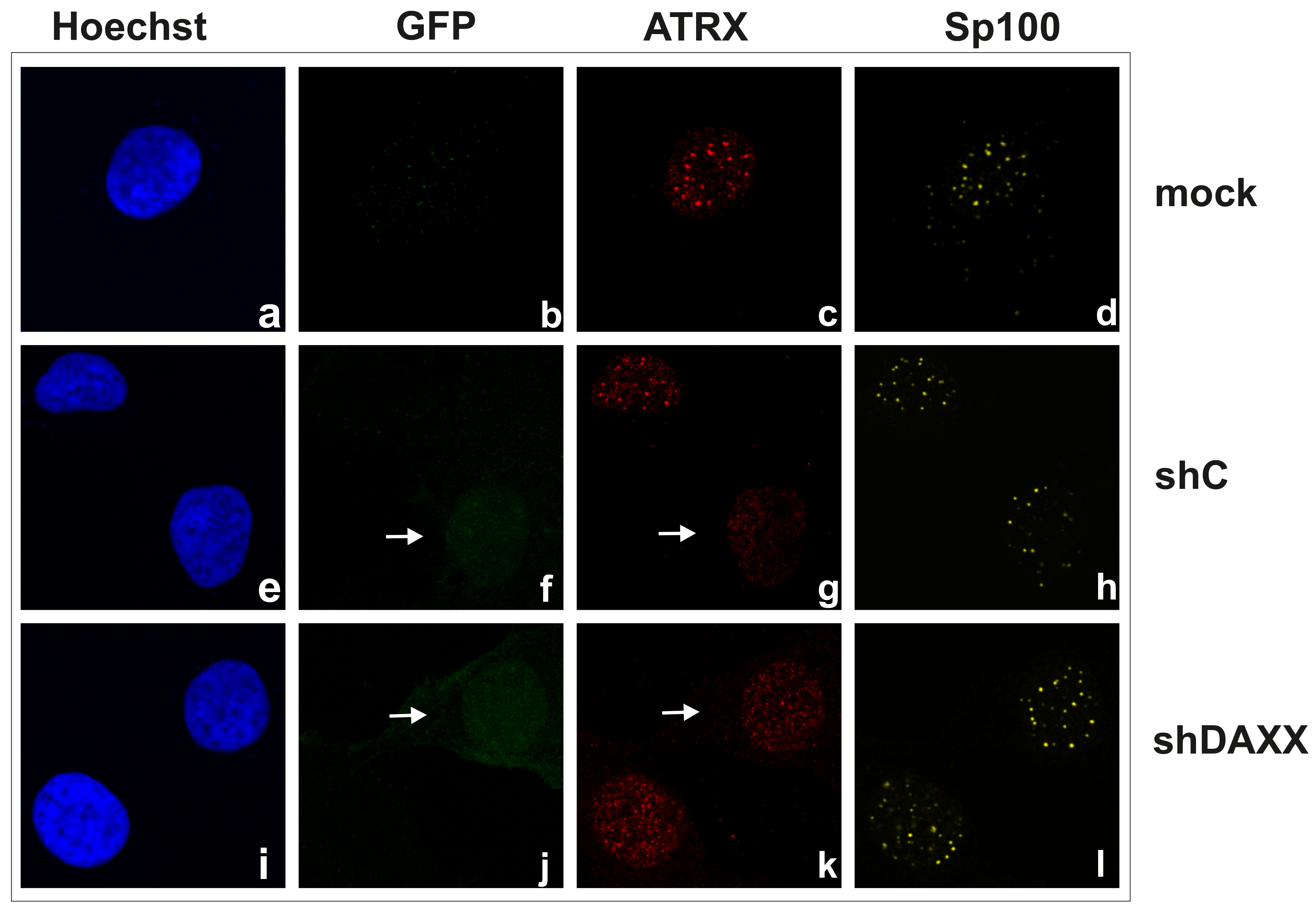

Supplement: Figure S6 — ATRX remains disperse in infected shDaxx cells. SLK cells mock treated (a–d), carrying knockdown shRNA vectors shC (e–h) or shDaxx (i–l) were infected by rKSHV.219 >(e–l) and immunostained with respective antibodies. The ND10 structure is detected by SP100 (for compatibility of antibody and secondary reagents). ATRX (g, arrow) is lost in infected shC (f, arrow) and seemingly also reduced in a Daxx-kd cell (j,k arrow) while Sp100 (h,l) remains in ND-10 structures. Localization of ATRX remains disperse after infection in shDaxx cells. (TIF) [file ppat.1003863.s006.tif]

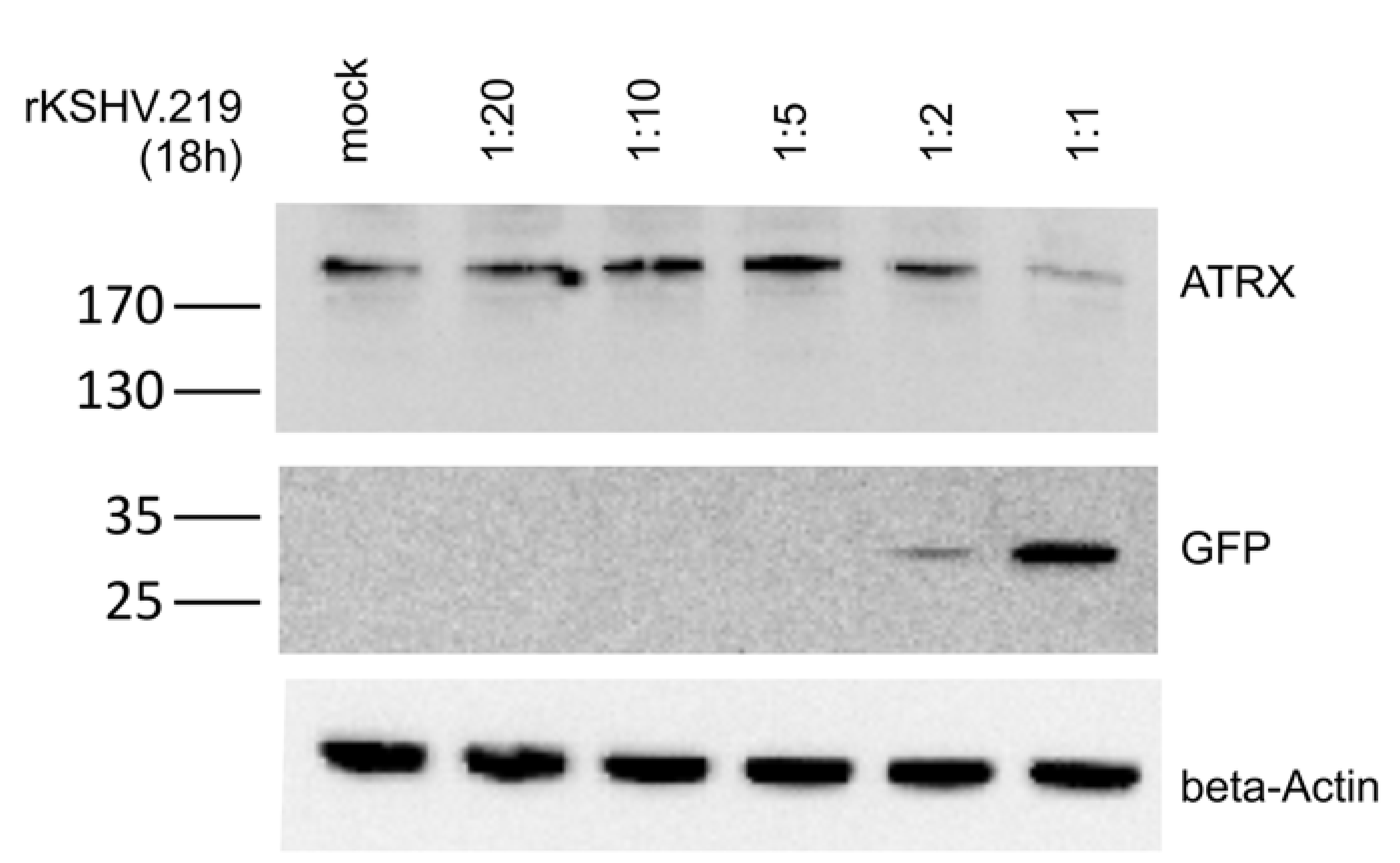

Supplement: Figure S7 — Disappearance of ATRX is dependent on virus amount. Equal numbers of SLK cells were seeded in 25 cm2 flasks; cells were infected with rKSHV.219 virus stock at the indicated dilutions or cultured, uninfected for 18 h; cells were harvested and expression of Actin, GFP, and ATRX was analyzed by immunoblotting. (TIF) [file ppat.1003863.s007.tif]

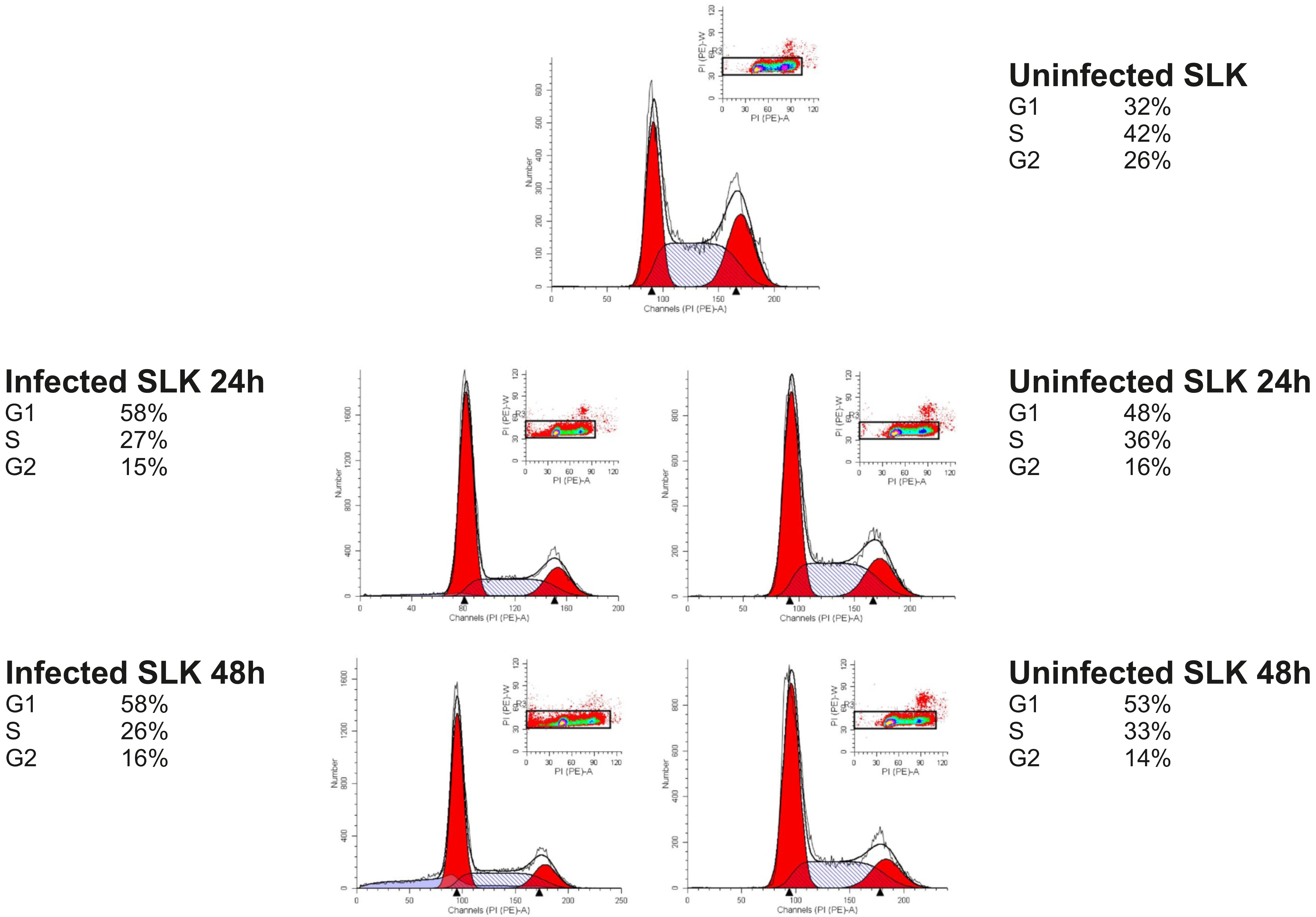

Supplement: Figure S8 — Infection by KSHV does not lead to cell cycle arrest in SLK cells. Equal numbers of Slk cells were seeded in 25 cm2 flasks; cells were infected with rKSHV.R219 or cultured uninfected. At the indicated time, cells were harvested and fixed in 80% ethanol. Flow cytometric cell cycle analysis was done after RNase treatment (50 µg/ml) and propidium iodide (20 µg/ml)staining on a BD LSR2. Single cells were selected by gating for FSC-area vs FSC-width and PI-area vs PI-with. Relative proportions of cells in G1, S or G2 were modeled with ModFitLT 3.3 for Windows (Verity Software House, Topsham, ME). (TIF) [file ppat.1003863.s008.tif]
